# Non-targeted metabolomic analysis for the comparative evaluation of volatile organic compounds in 20 globally representative cucumber lines

**DOI:** 10.3389/fpls.2022.1028735

**Published:** 2022-09-29

**Authors:** Hyo Eun Jo, Kihwan Song, Jeong-Gu Kim, Choong Hwan Lee

**Affiliations:** ^1^ Department of Bioscience and Biotechnology, Konkuk University, Seoul, South Korea; ^2^ Department of Bioresources Engineering, Sejong University, Seoul, South Korea; ^3^ Genomics Division, National Institute of Agricultural Sciences, Rural Development Administration, Jeonju, South Korea; ^4^ Research Institute for Bioactive-Metabolome Network, Konkuk University, Seoul, South Korea

**Keywords:** Metabolomics, cucumber, fruit flavor, volatile organic compounds, hydroperoxide lyase (HPL) and lipoxygenase (LOX) metabolisms

## Abstract

Volatile organic compounds (VOCs) are one of the main fruit-quality determinants in cucumber. Here, we investigated the differences in the VOC and primary metabolite composition among 20 representative cucumber lines. Results of non-targeted metabolomics revealed that the cucumber breeding line of the Korean group showed a unique VOC composition in the fruit peel compared to the other groups. Fruit-flesh VOCs significantly differed among Korean, European, and Thai fruits. The main cucumber flavor components, 2-hexenal, hexanal, 6-nonenal, 2,4-nonadienal, and 2,6-nonadienal, were lower in the Korean cucumber lines than in the others. Conversely, linoleic acid derivatives and α-linolenic acid, which are precursors of these VOCs, were abundant in Korean cucumber line. This suggests that the metabolism related to the characteristic flavor of cucumber are downregulated in Korean cucumber line. This study provides novel insights into the fruit flavor-associated metabolome in various cucumber lines.

## Introduction

Cucumber (*Cucumis sativus* L.) is a widely cultivated and consumed vegetable around the world (Tatlioglu, 2012; Che and Zhang, 2019). It is characterized by a fresh and distinct flavor (Hao et al., 2013; Che and Zhang, 2019; Zhang et al., 2021). However, consumer preferences for cucumbers varies vastly because of its distinct flavors. Because of this, numerous studies have been conducted to produce cucumbers with flavor characteristics suitable for different consumer preferences by enhancing the fruit quality through changes in cultivation methods (Guler et al., 2013; Li et al., 2019; Shan et al., 2020; Du et al., 2022a; Du et al., 2022b; Mi et al., 2022; Zhang et al., 2022).

In previous studies, 78 volatile organic compounds (VOCs) from different classes, including aldehydes, alcohols, alkanes, esters, and furans, have been identified in cucumbers (Hao et al., 2013). In addition, the main VOCs related to flavor in cucumbers have been identified. Based on the flavor threshold and contents, 2,6-nonadienal is the major aroma active compound among the various VOCs present in the cucumber fruit, thus being largely responsible for their characteristic flavor (Forss et al., 1962; Buescher and Buescher, 2001; Hao et al., 2013; Chen et al., 2015). Additionally, the C6 and C9 aldehydes derived from linoleic acid and synthesized by the lipoxygenase (LOX) and hydroperoxide lyase (HPL) are the major VOCs in cucumber (Stumpe et al., 2006; Chen et al., 2015; Shan et al., 2020). Particularly, C6 aldehydes contribute to the distinctive grassy, green flavors (Amaro et al., 2012; Chen et al., 2015), whereas C9 aldehydes contribute to the characteristic flower-like flavor (Chen et al., 2015). In addition, VOC content in the cucumber fruit is higher in the peel than in the flesh (Guler et al., 2013; Wei et al., 2016). Similarly, the cucumber fruit reportedly contain more aldehydes than other functional groups, such as alcohols, ketones, and terpenes (Guler et al., 2013; Wei et al., 2016). However, few studies have compared the peel and flesh VOC-composition among cucumber genotypes.

Metabolomics has significantly advanced our fundamental understanding of the natural variation in the metabolite composition among plants, as it provides information on the metabolic responses of living systems to changes in their genetic or environmental factors (Zhang et al., 2010; Matsuda et al., 2015; Sumner et al., 2015; Mun et al., 2021). In particular, non-targeted metabolomic approaches can be used for characterization and classification based on distinctive or characteristic metabolites in diverse plant species or genetic lines (Arbona et al., 2009; Carreno-Quintero et al., 2013; Díaz et al., 2016). Therefore, in this study, we have conducted a non-targeted metabolomics study to understand the differences in the flavor components among 20 cucumber breeding lines. Further, we aimed to determine how VOC metabolism differs between the fruit’s peel and flesh among different breeding lines. We performed the metabolomic analysis using gas chromatography time-of-flight mass spectrometry (GC-TOF-MS) and headspace-solid phase microextraction gas chromatography time-of-flight mass spectrometry (HS-SPME-GC-TOF-MS) platforms to compare VOCs and primary metabolites related to the flavor quality among different cucumber lines. In addition, the relative content of metabolites in different cucumber breeding lines were described in a metabolic pathway map aimed to enhance our understanding of the metabolism and molecular mechanisms associated to VOCs through the construction of a single pathway. Our results provide a solid theoretical basis for further research on the manipulation of cucumber flavor components in breeding programs focused on consumer preference-related traits.

## Materials and methods

### Chemicals and reagents

HPLC grade solvents were purchased from Fisher Scientific (Waltham, MA, USA). All standard compounds and analytical-grade reagents used were obtained from Sigma-Aldrich (St. Louis, MO, USA) and Junsei Chemicals (Tokyo, Japan).

### Sample information and preparation

**Figure 1:**
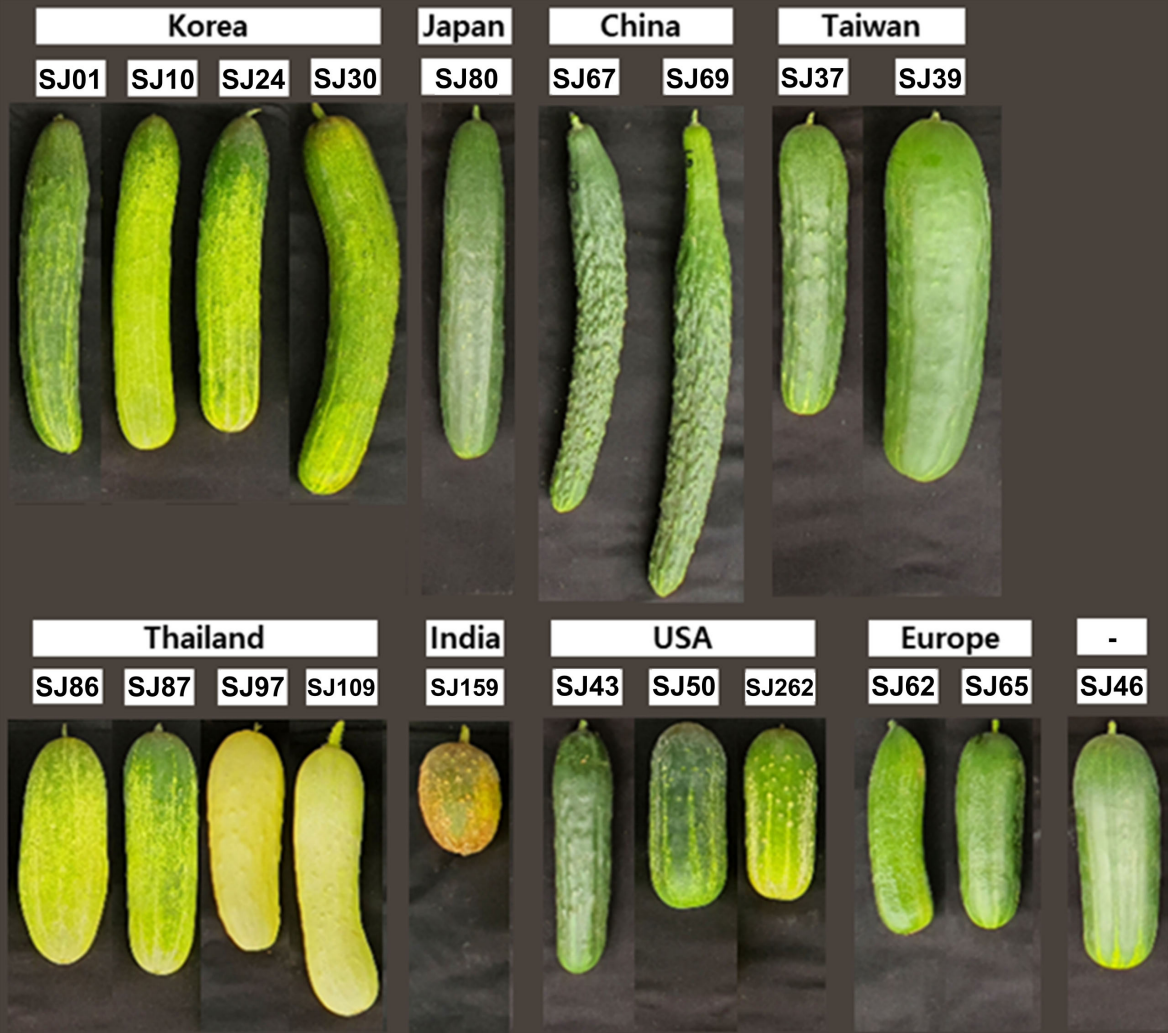
Immature fruits of the representative 20 cucumber breeding lines evaluated in this study.

To analyze the metabolome of the cucumber fruit, the following 20 cucumber breeding lines were used: SJ01, SJ10, SJ24, SJ30, SJ80, SJ67, SJ69, SJ37, SJ39, SJ86, SJ87, SJ97, SJ109, SJ159, SJ43, SJ50, SJ262, SJ62, SJ65, and SJ46 (**Figure 1**). Cucumbers were grown in soil inside a plastic greenhouse under sunlight from July 2020 to September 2020, in Anseong, Korea. Detailed sample information (line name, group, fruit length, fruit diameter, and fruit weight) for the 20 cucumber breeding lines is summarized in **Table S1**. Cucumber fruits were tagged on the day of anthesis, and were harvested 10 days after anthesis. After harvesting, fruits were stored at 17°C in a warehouse (Anseong, Korea) for a day. And fruits were transferred to a laboratory (Seoul, Korea) to perform metabolite analysis. Each cucumber fruit was washed with distilled water and divided into three segments based on length. Intermediate segments were used for metabolomic analysis. Peel and flesh were separated using a hand-held vegetable peeler. To analyze VOCs, fresh peel and flesh samples were ground into a powder under liquid nitrogen using a mortar and pestle. Powdered samples were stored at −80°C until VOC extraction. To extract primary metabolites, each sample was dried using a freeze dryer (Operon, Gimpo, Korea) for 5 days and then ground into a powder using a mortar and pestle. Dried powder samples were stored at −80°C until primary metabolite extraction. The analysis included three biological samples per breeding line.

### Sample extraction for primary metabolic profiling

Dried powder samples (100 mg) were extracted with 1 mL of 80% aqueous methanol, containing 1% v/v an internal standard solution (1 mg/mL of 2-chloro-L-phenylalanine in water), using an MM 400 mixer mill (Retsch^®^; Haan, Germany) at a frequency of 30 s^−1^ for 10 min, followed by sonication for 5 min at 4°C (Hettich Zentrifugen Universal 320; Tuttlingen, Germany). Extracted samples were centrifuged at 15,000 rpm for 10 min at 4°C, and the supernatants were filtered using 0.2 µm polytetrafluoroethylene (PTFE) syringe filters (Chromdisc, Daegu, Korea). The filtered supernatants were completely dried using a speed-vacuum concentrator (Biotron, Seoul, Korea). And 80% methanol was added to the dried extract to make a final concentration of 10,000 ppm (10 mg/mL) for analytical determination.

### Gas chromatography time-of-flight mass spectrometry (GC-TOF-MS) analysis for primary metabolites

Extracts in 80% MeOH was derivatized prior GC-TOF-MS analysis. For derivatization, 100 µL of the re-dissolved sample extract was collected in 1.5 mL Eppendorf tubes and completely dried using a speed-vacuum concentrator. Derivatization involved oximation and silylation. For oximation, 50 µL methoxyamine hydrochloride (20 mg/mL in pyridine) was added to the dried extract, and then the mixture was incubated for 90 min at 30°C. Silylation was performed by adding 50 µL of N-methyl-N-(trimethylsilyl) trifluoroacetamide to the mixture, followed by incubation for 30 min at 37°C. All samples were filtered using a 0.2 µm PTFE filter (Chromdisc, Daegu, Korea) prior to analytical determination. An aliquot of 1 µL sample was injected into the GC-TOF-MS instrument in the splitless mode. GC-TOF-MS analysis was performed using an Agilent 7890A GC system (Agilent Technologies, Palo Alto, CA, USA) coupled with an Agilent 7693 autosampler (Agilent Technologies) and Pegasus HT TOF-MS (LECO Corp., St. Joseph, MI, USA). Chromatographic separation was conducted using an Rtx-5MS column (30 m × 0.25 mm, 0.25 μm particle size; Restek Corp., St. Joseph, MI, USA) with helium as the carrier gas. Analytical methods and operation parameters used were described previously (Lee et al., 2016; Son et al., 2016). Analysis was performed on three biological and two analytical replicates.

### Headspace-solid phase microextraction gas chromatography time-of-flight mass spectrometry (HS-SPME-GC-TOF-MS) analysis for VOCs

To extract VOCs from the cucumber samples, the homogenized powder sample (5 g) was transferred into a 20 mL SPME glass vial (20 mL) and added 2 μL of a 20% NaCl/Octanal (98:2, v/v) solution. For volatile collection, headspace-solid phase microextraction (HS-SPME) of the VOCs was performed using a 50/30 µm divinylbenzene/carboxen™/polydimethylsiloxane (DVB-CAR-PDMS) StableFlex™ fiber (Sigma Aldrich, St. Louis, MO, USA). The SPME fiber, preheated to 270°C for 1 min, was injected into the SPME vial and exposed to the headspace for 20 min at 60°C. The fiber was then introduced into the injector port of a GC-MS instrument (7890A GC-5975C MSD; Agilent) equipped with a DB-FFAP column (30 m × 0.25 mm, 0.25 µm film; J&W Scientific, Folsom, CA, USA). The injector (in splitless mode) temperature was set at 250°C, and extraction of VOCs was performed by exposing the SPME fiber to the headspace of the sample supernatants for 30 min at 37°C. Oven temperature was initially set to 50°C for 2 min and then increased to 300°C at a rate of 10°C min^−1^. Upon reaching 300°C, the temperature was held at that point for 3 min. The temperature of the transfer line was set at 240°C. After extraction, the fiber was removed from the holder and desorbed at the GC port for 1 min at 270°C. The analytical methods and operational parameters used for VOC analysis were based on our previous study (Jo et al., 2022). Sample analysis was performed using two analytical replicates.

### Data processing and multivariate statistical analysis

Raw data obtained by GC-TOF-MS and HS-SPME-GC-TOF-MS were converted to NetCDF (*. cdf) format using LECO ChromaTOF software (version 4.44). After conversion, the NetCDF files were processed using the metAlign software package for peak detection, retention time correction, and alignment. Thereafter, the processed data were used by SIMCA-P+ 12.0 (Umetrics, Umea, Sweden) for principal component analysis (PCA) and partial least squares discriminant analysis (PLS-DA). To select significantly different metabolites among samples, variable importance in the projection (VIP > 0.7) values were based on a PLS-DA score plot. In addition, a significance test (p < 0.05) for differences between biological and analytical replicates was performed by analysis of variance (ANOVA) and Duncan’s multiple range tests using the PASW Statistics 18 software (SPSS, Inc., Chicago, IL, USA). Selected metabolites were tentatively identified by comparison of various parameters, including mass fragment patterns, retention times, and mass spectra of data for standard compounds under the same conditions obtained from published papers and commercial databases, such as the National Institute of Standards and Technology (NIST) Library (version 2.0, 2011, FairCom, Gaithersburg, MD, USA) and Wiley 9.

## Results

### Analysis of VOCs in 20 cucumber breeding lines

**Figure 2:**
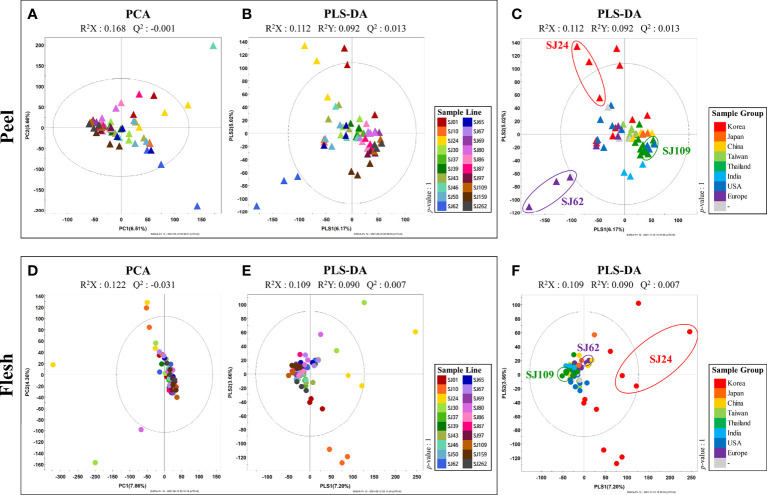
PCA and PLS-DA score plots of 20 cucumber breeding lines of peel **(A–C)** and flesh **(D–F)** based on HS-SPME-GC-TOF-MS data set. **(A, B, D, E)** indicate the color of sample line. **(C, F)** indicate the color of sample group. 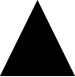, peel; 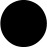, flesh.

Cucumber peel and flesh VOC profiles of 20 different breeding-lines were examined using HS-SPME-GC-TOF-MS. To evaluate the variation in VOCs profile among the 20 cucumber breeding lines, a multivariate analysis was performed. Although the PCA models obtained from HS-SPME-GC-TOF-MS analysis displayed clustering patterns for each line (**Figures 2A, D**), it was difficult to identify a clearly distinct pattern per breeding line due to the enormous diversity among the samples (**Figures 2A, D**). Therefore, PLS-DA was performed to confirm the differences among breeding lines in more detail (**Figures 2B, E**). PLS-DA models obtained from HS-SPME-GC-TOF-MS analysis revealed that the cucumber fruit (i.e., peel and flesh) clustered together depending on the geographical group to which they belong (**Figures 2C, F**). Thus, with regard to peel, the “European group” (“SJ62” and “SJ65”) was separated from other groups across PLS1 (6.17%). In turn, the “Korean group” (“SJ01,” “SJ10,” “SJ24,” and “SJ30”) was distinguished from other groups across PLS2, (5.02%), while the “Thai group” (“SJ86,” “SJ87,” “SJ97,” and “SJ109”) was significantly different from the Korean and the European groups (**Figure 2C**). To investigate distinctive VOCs of the different groups of cucumbers, we selected significantly discriminated metabolites based on the VIP values (>0.7) derived from the PLS-DA model. In all, 21 VOCs, including 11 aldehydes, 4 alcohols, 2 acids, 1 furan, 1 sesquiterpenoid were determined as significantly distinct among the peels of the different breeding lines in this study (**Table S2**). The relative abundance value of these 21 VOCs were displayed on a **Table S4**. Each column indicated an average of the relative abundance value of each cucumber line. In particular, the levels of several aldehydes and alcohols, including hexanal, 6-nonenal, benzaldehyde, pentanal, 2,4-nonadienal, and 1-octen-3-ol, were present in clearly low concentrations in the Korean and European groups. In contrast, Thai cucumbers showed higher levels of these compounds than the former groups (**Table S4**).

In turn, the analysis of cucumber flesh allowed the identification of a more discriminant, line-dependent VOC-profiling trend than the analysis of the peel. Most cucumber lines were clustered together in the middle, but the “Korean group” cucumbers (“SJ01,” “SJ10,” “SJ24,” and “SJ30”) were specifically distinguished from other groups by PLS1 (7.20%) (**Figure 2F**). To investigate the distinctive VOCs of the different groups of breeding lines, we selected significantly discriminated metabolites based on VIP value (>0.7) derived from the PLS-DA model. Thus, 19 volatile compounds, including 10 aldehydes, 6 alcohols, 1 acid, 1 furan, and 1 sesquiterpenoid, were found to be significantly discriminant among the flesh of different cucumber breeding lines (**Table S3**). The relative abundance value of these 19 VOCs were displayed on a **Table S5**. Each column indicated an average of the relative abundance value of each cucumber line. Most aldehydes, including 2,6-nonadienal, propanal, pentanal, 2,4-nonadienal, 2-octenal, tetradecanal, hexadecanal, hexanal, and 2-hexanal, were present in relatively low levels in cucumbers of the Korean group, compared to the others. In contrast, several alcohols, such as 3-nonen-1-ol, 3,6-nonadien-ol, and (6*Z*)-nonen-1-ol, were detected at relatively high levels in the Korean cucumbers. In addition, nonanoic acid, 2-ethylfuran, and β-ionone were clearly detected at low levels in the Korean cucumbers, relative to the other groups (**Table S5**).

### Comparative metabolomics study of Korean, European, and Thai cucumbers

Because of the enormous diversity of the samples, the analysis of VOCs presents in 20 cucumber breeding lines showed great difficulty to identify a clearly distinguished pattern per breeding line. Therefore, we tried to narrow down the number of samples into three groups: Korean, European, and Thai, which were the ones most clearly distinguished from all other groups, as per multivariate statistical analysis from HS-SPME-GC-TOF-MS of 20 breeding lines. Specifically, the cucumber lines (“SJ24,” “SJ62,” and “SJ109”) were selected, as they were farthest apart from the rest in the PLS-DA model. These three lines belong to the Korean, European, and Thai groups, respectively. By analyzing the three most strikingly different cucumbers as per multivariate statistical analysis, we tried to identify the VOC metabolic pathways responsible for the observed differences in cucumber flavor. Primary metabolites and VOC profiles from peel and flesh of three different cucumber lines, namely, “SJ24”, “SJ62”, and “SJ109,” were examined using GC-TOF-MS and HS-SPME-GC-TOF-MS. Variations in primary metabolites and VOC profiles between the peel and flesh of the three lines were examined based on multivariate analysis of the respective datasets.

**Figure 3:**
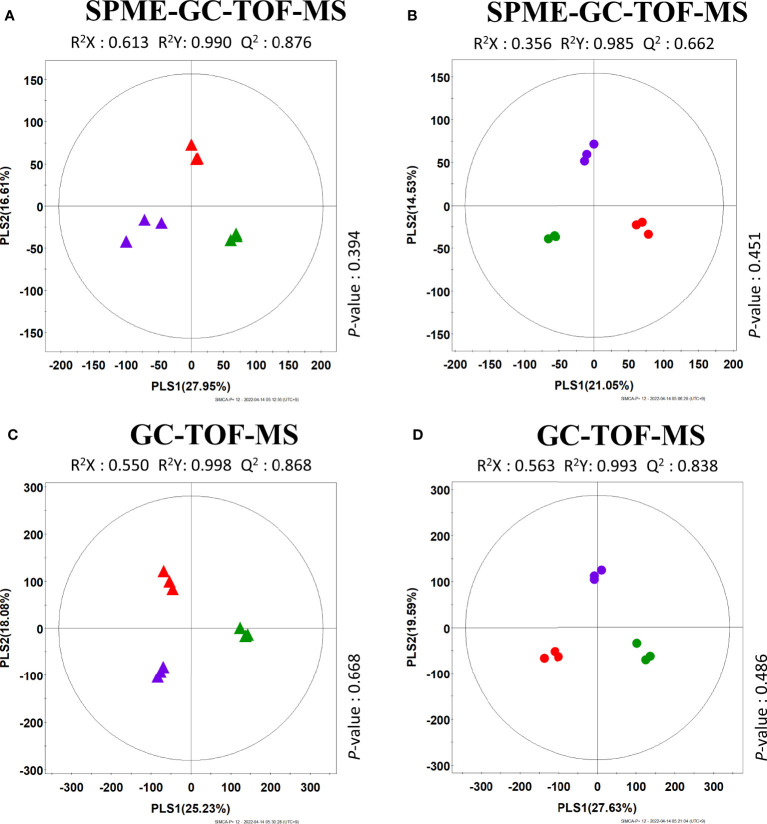
PLS-DA score plots of different three cucumbers based on SPME-GC-TOF-MS **(A, B)** and GC-TOF-MS **(C, D)** data set. **(A, C)** are the results of analyzing different peel data. **(B, D)** are the results of analyzing different flesh data. Different sample symbolized as: Peel (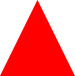: SJ24 (Korea group), 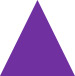: SJ62 (Europe group), 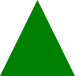: SJ109 (Thailand group)), flesh (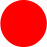: SJ24 (Korea group), 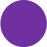: SJ62 (Europe group), 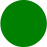: SJ109 (Thailand group)).

The results revealed that the three cucumbers could be distinguished from each other and clustered depending on the corresponding breeding line (**Figure S1**). The PLS-DA score plot also showed a pattern similar to that of the PCA score plot (**Figure 3**). To investigate the distinctive metabolites among the three cucumbers, we selected significantly discriminated metabolites based on VIP value (>0.7), derived from the PLS-DA model.

Thirty-two significantly discriminant metabolites, including 11 amino acids, 4 organic acids, 7 carbohydrates, 3 fatty acids, hydroxylamine, uracil, and 5 non-identified compounds were selected using the PLS-DA model based on peel GC-TOF-MS datasets (**Table S6**). In addition, 22 volatiles, including 11 aldehydes, 6 alcohols, 1 acid, 1 furan, 1 sesquiterpenoid, and 2 non-identified compounds were determined as significantly discriminant, based on the PLS-DA model for the HS-SPME-GC-TOF-MS datasets (**Table S7**). Meanwhile, in the fruit flesh, 35 significantly discriminant metabolites, including 13 amino acids, 3 organic acids, 9 carbohydrates, 3 fatty acids, 2-ethylfuran, β-ionone, and octanoic acid were selected using the PLS-DA model based on GC-TOF-MS datasets (**Table S8**). In addition, 17 volatiles, including 9 aldehydes, 4 alcohols, 1 furan, and 1 sesquiterpenoid compounds were determined to be significantly discriminant based on the PLS-DA model for the HS-SPME-GC-TOF-MS datasets (**Table S9**).

**Figure 4:**
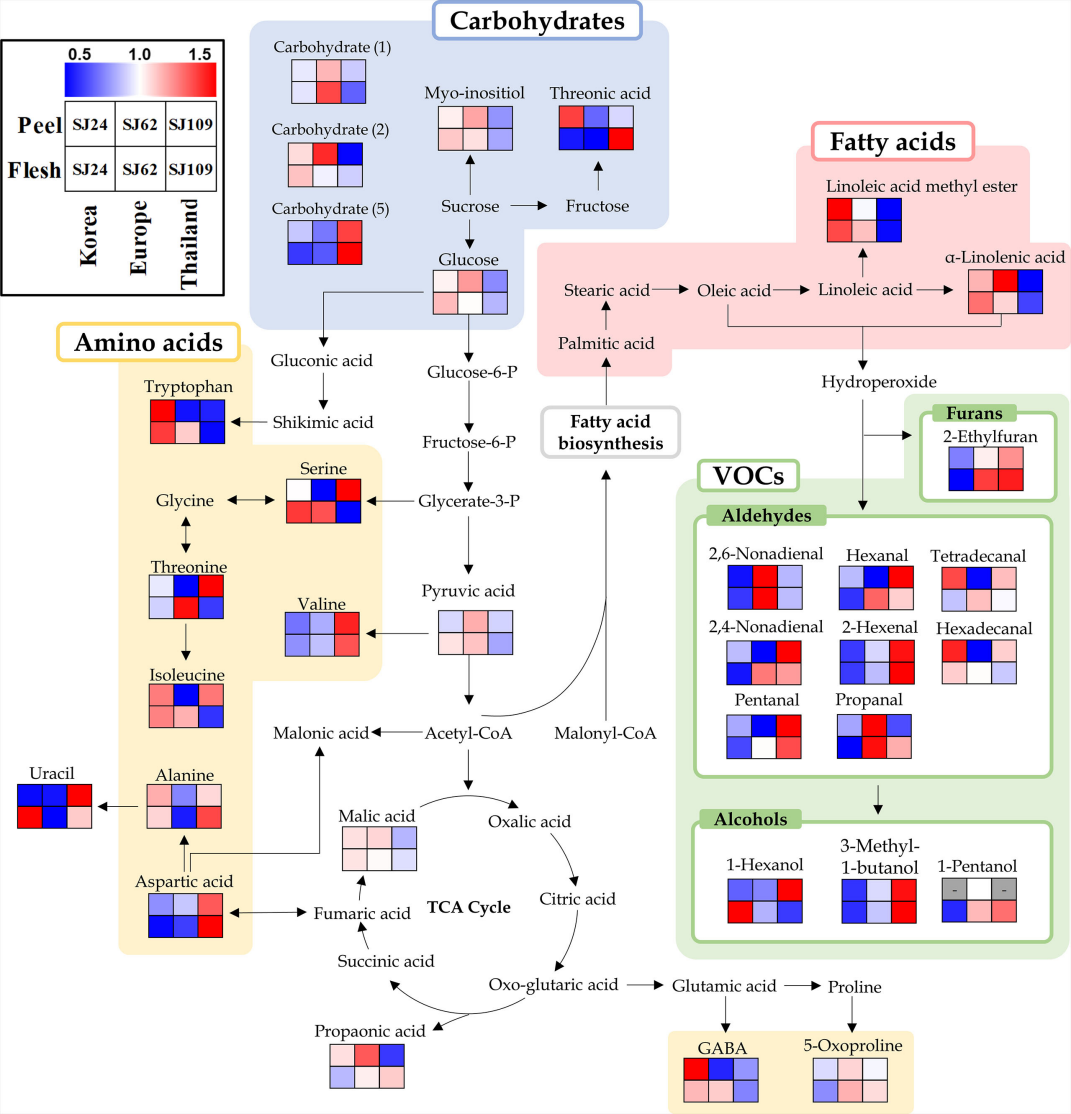
Schematic diagram of the biosynthetic pathway and relative content of metabolites in SJ24, SJ62, and SJ109 cucumbers. The pathway was modified from the Kyoto Encyclopedia of Genes and Genomes (KEGG) database (http://www.genome.jp/kegg/). The colored squares (blue-to-red) represent the relative abundance.

Moreover, we performed metabolic pathway analysis integrating primary and VOC metabolism to understand the biosynthesis of VOCs in cucumber fruits. Thirty-three metabolites selected as significantly discriminant in both peel and flesh were displayed on a metabolic pathway using a heat scale box (**Figure 4**). The furan and aldehydes located at the beginning of the VOCs synthesis pathway were relatively low in “SJ24” (Korean group) than in “SJ62” (European group) or in “SJ109” (Thai group). Particularly, C6 (hexanal and 2-hexanal) and C9 aldehydes (2,6-nonadienal and 2,4-nonadienal) showed clearly low patterns in “SJ24” than in other lines. These compounds are known to be the main VOCs in cucumber fruits. In contrast, linoleic acid derivatives and α-linolenic acid, which are precursors of VOCs, were found in relatively high concentration in “SJ24.” This trend was the same for both peel and flesh.

Neither amino acid nor carbohydrate metabolism showed any distinct trend in any of the cucumber samples. With respect to carbohydrate metabolism, glucose, myo-inositol, carbohydrate (1), and carbohydrate (2) were relatively lower in “SJ109” of the Thai group than in any other group. Conversely, carbohydrate (5) exhibited the opposite pattern. In turn, threonic acid was relatively highly abundant in peels of “SJ24” (Korea group), whereas in the flesh, it was higher in “SJ109” (Thai group). As for amino acid metabolism, valine and aspartic acid showed a high relative abundance in “SJ109” (Thai group), while tryptophan and GABA showed a higher relative abundance in “SJ24” (Korean group) than in “SJ62” (European group) or “SJ109” (Thai group).

## Discussion

Fruit flavor characteristics are important components of cucumber fruit quality. Cucumber fruit is well known for its distinct flavor, which is crucial for evaluating fruit quality (Palma-Harris et al., 2001). In a previous study, 78 VOCs related to fruit flavor were identified in cucumber fruits, including aldehydes, alcohols, esters, alkanes, and furans (Hao et al., 2013). Aldehydes accounted for a large proportion of these VOCs (Chen et al., 2015). Furthermore, C6 and C9 aldehydes are the most important compounds for fruit and vegetable flavor (Ligor and Buszewski, 2008; Wang et al., 2010; Shen et al., 2014; Li et al., 2019). Specifically, C6 and C9 aldehydes contribute to the distinctive grassy and green, and flower-like flavors, respectively (Ligor and Buszewski, 2008; Amaro et al., 2012; Chen et al., 2015). The main volatile compound in cucumber, 2,6-nonadienal, which has cucumber-like flavor, is a C9 aldehyde (Buescher and Buescher, 2001; Chen et al., 2015).

In this study, we performed VOC profiling to compare fruit flavor quality among 20 different cucumber breeding lines that are the main representative types of cucumbers consumed worldwide. Multivariate analysis of both peel and flesh data revealed that sampled cucumbers were clustered depending on the country of origin (**Figures 2C, F**). Especially with regard to the peel, European (“SJ62” and “SJ65”) and Korean (“SJ01,” “SJ10,” “SJ24,” and “SJ30”) cucumbers were separated from other groups across PLS1 and PLS2 (**Figure 2C**). In addition, the Thai group of breeding lines (“SJ86,” “SJ87,” “SJ97,” and “SJ109”) were separated farthest from the breeding lines in the Korean and European groups (**Figure 2C**). Several C6 and C9 aldehydes (hexanal, 2,4-nonadienal, and 6-nonenal) were present at relatively low levels in the cucumbers of the Korean and European groups. In contrast, cucumbers in the Thai group showed higher levels of these compounds than any other cucumbers. Similarly, 1-octen-3-ol showed a pattern similar to that of these compounds. A characteristic phenomenon was observed in the flesh of cucumbers of the Korean group (“SJ01,” “SJ10,” “SJ24,” and “SJ30”), which distinguished this from all other groups by PLS1 (**Figure 2F**). The levels of most aldehydes, including propanal, pentanal, 2-octenal, tetradecanal, hexadecanal, C6 aldehydes (hexanal and 2-hexanal), and C9 aldehydes (2,6-nonadienal and 2,4-nonadienal), were relatively low in cucumbers of the Korean group. However, several C9 alcohols, such as 3-nonen-1-ol, 3,6-nonadien-ol, and (6Z)-nonen-1-ol, were more abundant in cucumbers of this group than in those of any other group. In addition, nonanoic acid, 2-ethylfuran, and β-ionone were present in low levels in the Korean group, compared with other groups (**Figure 3B**). In a previous study, C6 aldehydes were reportedly related to a grassy, green flavor, while C9 aldehydes were found associated to a flower-like flavor (Chen et al., 2015). In addition, propanal has a stimulate flavor, and pentanal has a fresh flavor (Chen et al., 2015). In turn, 2-ethylfuran contributes to a fruity flavor, and β-ionone affects the flower-like flavor (Wei et al., 2016). Therefore, it can be inferred that cucumbers of the Korean group have relatively lower levels of distinct flavors derived from C6 and C9 aldehydes in both peel and flesh, than those in other groups.

Volatiles of the C6 and C9 types are produced *via* the lipoxygenase (LOX) and hydroperoxide lyase (HPL) pathways (Sivankalyani et al., 2017; Li et al., 2019; Shan et al., 2020). They are synthesized from polyunsaturated fatty acids, such as linoleic acid and α-linolenic acid, by LOX and HPL enzymes (Boonprab et al., 2003; Boonprab et al., 2006; Mosblech et al., 2009). Further, LOX and HPL are key enzymes in the oxylipin pathway and are encoded by multiple genes (Schwab et al., 2008). Stereospecific oxidation of VOC precursors, such as linoleic acid and α-linolenic acid, is catalyzed by LOX. Plant LOX enzymes are classified as 9-LOX or 13-LOX according to their positional specificity of fatty acid oxygenation in the case of a C18 fatty acid of the hydrocarbon backbone (Mosblech et al., 2009). The hydroperoxides synthesized using 9-LOX were cleaved into C9 aldehydes by 9-HPL. Meanwhile, hydroperoxides produced by 13-LOX are cleaved into C6 aldehydes by 13-HPL (Matsui et al., 2006; Shan et al., 2020).

To understand the synthesis of VOCs affecting fruit flavor, additional primary metabolome analysis was performed on the three cucumber lines (SJ24, SJ62, and SJ109), which multivariate analysis revealed as the ones farthest apart. 2-Ethylfuran, C6 aldehydes (hexanal and 2-hexenal), and C9 aldehydes (2,6-nonadienal and 2,4-nonadienal) located at the beginning of the VOCs synthesis pathway showed clearly lower relative contents in “SJ24” (Korean group) than in “SJ62” (Europe group) or in “SJ109” (Thai group). However, linoleic acid derivatives and α-linolenic acid, which are precursors of these VOCs, were found to be relatively higher in “SJ24” than in the other two lines (**Figure 4**). The opposite patterns of C6 and C9 aldehydes and fatty acids in all precursors of VOCs were observed in both cucumber peel and flesh. Therefore, we inferred that the process of synthesis of C6 and C9 aldehydes from fatty acids is downregulated in the Korean cucumber lines, compared to the other groups of lines, likely due to the lesser LOX and HPL activities, whereby, the Korean cucumber lines showed relatively less flavor characteristics.

## Conclusion

We analyzed the differences in volatiles of 20 cucumber breeding lines. Lines of the Korean, European, and Thai groups showed specific peel-VOC patterns that clearly distinguished them from other groups. Hexanal, 6-nonenal, and 2,4-nonadienal showed a pattern of relatively higher concentrations in the Thai group, in contrast with the lower concentrations in the Korean and European breeding lines. With respect to the flesh, the Korean group differed from others in that it showed low levels of 2,6-nonadienal, 2,4-nonadienal, hexanal, and 2-hexenal. However, linoleic acid methyl ester, and α-linolenic acid, which are precursors of volatile organic compounds, were found to be relatively higher in the Korean cucumbers than in those of other groups both in the peel and flesh. This result suggests that the LOX and HPL associated synthesis of volatiles from fatty acids are downregulated in the Korean cucumber lines.

This study presents profiling information on VOC-related quality characteristics that greatly affect consumer preferences for 20 cucumber breeding lines. It was obtained by the non-targeted metabolomic approach. In addition, we found that the Korean cucumber lines evaluated herein show relatively less intense flavor characteristics than other lines. Thus, we propose the Korean cucumber line could have an advantage for consumers who are sensitive and dislike the strong flavor of cucumber fruit.

## Data availability statement

The original contributions presented in the study are included in the article/**Supplementary Material**. Further inquiries can be directed to the corresponding authors.

## Author contributions

HJ, KS, J-GK, and CL conceived of and designed the study. KS prepared the plant materials. HJ performed metabolite profiling, data processing, and statistical analysis. HJ wrote the manuscript. KS, J-GK, and CL revised the manuscript. All authors have contributed to the manuscript and approved the submitted version.

## Acknowledgments

This research was carried out with the support of Konkuk University research support program and RDA (Rural Development Administration, Republic of Korea) “Cooperative Research Program for National Agricultural Genome Program (Project No. PJ01343202)”.

## Conflict of interest

The authors declare that the research was conducted in the absence of any commercial or financial relationships that could be construed as a potential conflict of interest.

## Publisher’s note

All claims expressed in this article are solely those of the authors and do not necessarily represent those of their affiliated organizations, or those of the publisher, the editors and the reviewers. Any product that may be evaluated in this article, or claim that may be made by its manufacturer, is not guaranteed or endorsed by the publisher.

## References

[B1] Amaro A. L. Beaulieu J. C. Grimm C. C. Stein R. E. Almeida D. P. F. (2012). Effect of oxygen on aroma volatiles and quality of fresh-cut cantaloupe and honeydew melons. Food Chem. 130, 49–57. doi: 10.1016/j.foodchem.2011.06.052

[B2] Arbona V. Iglesias D. J. Talón M. Gómez-Cadenas A. (2009). Plant phenotype demarcation using nontargeted LC-MS and GC-MS metabolite profiling. J. Agric. Food Chem. 57, 7338–7347. doi: 10.1021/jf9009137 19639992

[B3] Boonprab K. Matsui K. Akakabe Y. Yoshida M. Yotsukura N. Chirapart A.. (2006). Formation of aldehyde flavor (n-hexanal, 3Z-nonenal and 2E-nonenal) in the brown alga, *Laminaria angustata* . J. Appl. Phycol. 18, 409–412. doi: 10.1007/s10811-006-9038-6

[B4] Boonprab K. Matsuia K. Yoshida M. Akakabe Y. Chirapart A. Kajiwara T. (2003). C6-aldehyde formation by fatty acid hydroperoxide lyase in the brown alga *Laminaria angustata* . Z. Für Naturforsch. C 58, 207–214. doi: 10.1515/znc-2003-3-412 12710730

[B5] Buescher R. H. Buescher R. W. (2001). Production and stability of (E, z)-2, 6-nonadienal, the major flavor volatile of cucumbers. J. Food Sci. 66, 357–361. doi: 10.1111/j.1365-2621.2001.tb11346.x

[B6] Carreno-Quintero N. Bouwmeester H. J. Keurentjes J. J. B. (2013). Genetic analysis of metabolome–phenotype interactions: from model to crop species. Trends Genet. 29, 41–50. doi: 10.1016/j.tig.2012.09.006 23084137

[B7] Chen S. Zhang R. Hao L. Chen W. Cheng S. (2015). Profiling of volatile compounds and associated gene expression and enzyme activity during fruit development in two cucumber cultivars. PloS One 10, 1–22. doi: 10.1371/journal.pone.0119444 PMC4370779 25799542

[B8] Che G. Zhang X. (2019). Molecular basis of cucumber fruit domestication. Curr. Opin. Plant Biol. 47, 38–46. doi: 10.1016/j.pbi.2018.08.006 30253288

[B9] Díaz R. Gallart-Ayala H. Sancho J. V. Nuñez O. Zamora T. Martins C. P. B.. (2016). Told through the wine: A liquid chromatography–mass spectrometry interplatform comparison reveals the influence of the global approach on the final annotated metabolites in non-targeted metabolomics. J. Chromatogr. A 1433, 90–97. doi: 10.1016/j.chroma.2016.01.010 26795279

[B10] Du X. Davila M. Williams C. Weng Y. (2022a). Fresh cucumber fruit physicochemical properties, consumer acceptance, and impact of variety and harvest date. ACS Food Sci. Technol. 2, 616–629. doi: 10.1021/acsfoodscitech.1c00433

[B11] Du X. Routray J. Williams C. Weng Y. (2022b). Association of refreshing perception with volatile aroma compounds, organic acids, and soluble solids in freshly consumed cucumber fruit. ACS Food Sci. Technol. 2 (9), 1495–1506. doi: 10.1021/acsfoodscitech.2c00195

[B12] Forss D. A. Dunstone E. A. Ramshaw E. H. Stark W. (1962). The flavor of cucumbers. J. Food Sci. 27, 90–93. doi: 10.1111/j.1365-2621.1962.tb00064.x

[B13] Guler Z. Karaca F. Yetisir H. (2013). Volatile compounds in the peel and flesh of cucumber (*cucumis sativus* l.) grafted onto bottle gourd (*Lagenaria siceraria*) rootstocks. J. Hortic. Sci. Biotechnol. 88, 123–128. doi: 10.1080/14620316.2013.11512945

[B14] Hao L. Chen S. Wang C. Chen Q. Wan X. Shen X.. (2013). Aroma components and their contents in cucumbers from different genotypes. J. Northwest A F Univ. Sci. Ed. 41, 139–146. Available at: https://www.cabdirect.org/cabdirect/abstract/20133256240

[B15] Jo H. E. Son S. Y. Lee C. H. (2022). Comparison of metabolome and functional properties of three Korean cucumber cultivars. Front. Plant Sci. 13. doi: 10.3389/fpls.2022.882120 PMC9051474 35498687

[B16] Lee M. Y. Singh D. Kim S. H. Lee S. J. Lee C. H. (2016). Ultrahigh pressure processing produces alterations in the metabolite profiles of panax ginseng. Molecules 21, 1–16. doi: 10.3390/molecules21060816 PMC6273588 27338333

[B17] Ligor T. Buszewski B. (2008). Single-drop microextraction and gas chromatography–mass spectrometry for the determination of volatile aldehydes in fresh cucumbers. Anal. Bioanal. Chem. 391, 2283–2289. doi: 10.1007/s00216-008-2098-5 18415084

[B18] Li X. Sun Y. Wang X. Dong X. Zhang T. Yang Y.. (2019). Relationship between key environmental factors and profiling of volatile compounds during cucumber fruit development under protected cultivation. Food Chem. 290, 308–315. doi: 10.1016/j.foodchem.2019.03.140 31000051

[B19] Matsuda F. Nakabayashi R. Yang Z. Okazaki Y. Yonemaru J. Ebana K.. (2015). Metabolome-genome-wide association study dissects genetic architecture for generating natural variation in rice secondary metabolism. Plant J. 81, 13–23. doi: 10.1111/tpj.12681 25267402 PMC4309412

[B20] Matsui K. Minami A. Hornung E. Shibata H. Kishimoto K. Ahnert V.. (2006). Biosynthesis of fatty acid derived aldehydes is induced upon mechanical wounding and its products show fungicidal activities in cucumber. Phytochemistry 67, 649–657. doi: 10.1016/j.phytochem.2006.01.006 16497344

[B21] Mi S. Zhang X. Wang Y. Ma Y. Sang Y. Wang X. (2022). Effect of different fertilizers on the physicochemical properties, chemical element and volatile composition of cucumbers. Food Chem. 367, 130667. doi: 10.1016/j.foodchem.2021.130667 34339981

[B22] Mosblech A. Feussner I. Heilmann I. (2009). Oxylipins: Structurally diverse metabolites from fatty acid oxidation. Plant Physiol. Biochem. 47, 511–517. doi: 10.1016/j.plaphy.2008.12.011 19167233

[B23] Mun H. I. Kwon M. C. Lee N. R. Son S. Y. Song D. H. Lee C. H. (2021). Comparing metabolites and functional properties of various tomatoes using mass spectrometry-based metabolomics approach. Front. Nutr. 8. doi: 10.3389/fnut.2021.659646 PMC8060453 33898504

[B24] Palma-Harris C. McFeeters R. F. Fleming H. P. (2001). Solid-phase microextraction (SPME) technique for measurement of generation of fresh cucumber flavor compounds. J. Agric. Food Chem. 49, 4203–4207. doi: 10.1021/jf010182w 11559111

[B25] Schwab W. Davidovich-Rikanati R. Lewinsohn E. (2008). Biosynthesis of plant-derived flavor compounds. Plant J. 54, 712–732. doi: 10.1111/j.1365-313X.2008.03446.x 18476874

[B26] Shan N. Gan Z. Nie J. Liu H. Wang Z. Sui X. (2020). Comprehensive characterization of fruit volatiles and nutritional quality of three cucumber (*Cucumis sativus* l.) genotypes from different geographic groups after bagging treatment. Foods 9 (3), 294. doi: 10.3390/foods9030294 PMC7143270 32150913

[B27] Shen J. Y. Wu L. Liu H. R. Zhang B. Yin X. R. Ge Y. Q.. (2014). Bagging treatment influences production of C6 aldehydes and biosynthesis-related gene expression in peach fruit skin. Molecules 19, 13461–13472. doi: 10.3390/molecules190913461 25178066 PMC6271678

[B28] Sivankalyani V. Maoz I. Feygenberg O. Maurer D. Alkan N. (2017). Chilling stress upregulates α-linolenic acid-oxidation pathway and induces volatiles of C6 and C9 aldehydes in mango fruit. J. Agric. Food Chem. 65, 632–638. doi: 10.1021/acs.jafc.6b04355 28075566

[B29] Son S. Y. Kim N. K. Lee S. Singh D. Kim G. R. Lee J. S.. (2016). Metabolite fingerprinting, pathway analyses, and bioactivity correlations for plant species belonging to the cornaceae, fabaceae, and rosaceae families. Plant Cell Rep. 35, 1917–1931. doi: 10.1007/s00299-016-2006-y 27344340

[B30] Stumpe M. Bode J. Göbel C. Wichard T. Schaaf A. Frank W.. (2006). Biosynthesis of C9-aldehydes in the moss physcomitrella patens. Biochim. Biophys. Acta (BBA)-Molecular Cell Biol. Lipids 1761, 301–312. doi: 10.1016/j.bbalip.2006.03.008 16630744

[B31] Sumner L. W. Lei Z. Nikolau B. J. Saito K. (2015). Modern plant metabolomics: advanced natural product gene discoveries, improved technologies, and future prospects. Nat. Prod. Rep. 32, 212–229. doi: 10.1039/c4np00072b 25342293

[B32] Tatlioglu T. (2012). “Cucumber: Cucumis sativus l,” in Genetic improvement of vegetable crops. Eds. Kalloo G. Bergh B. O. (Oxford: Newnes), 197–234.

[B33] Wang Y. Yang C. Liu C. Xu M. Li S. Yang L.. (2010). Effects of bagging on volatiles and polyphenols in “Wanmi” peaches during endocarp hardening and final fruit rapid growth stages. J. Food Sci. 75 (9), S455–S460. doi: 10.1111/j.1750-3841.2010.01817.x 21535618

[B34] Wei G. Tian P. Zhang F. Qin H. Miao H. Chen Q.. (2016). Integrative analyses of nontargeted volatile profiling and transcriptome data provide molecular insight into VOC diversity in cucumber plants (*Cucumis sativus*). Plant Physiol. 172, 603–618. doi: 10.1104/pp.16.01051 27457123 PMC5074635

[B35] Zhang J. Feng S. Yuan J. Wang C. Lu T. Wang H.. (2021). The formation of fruit quality in *Cucumis sativus* l. Front. Plant Sci. 12. doi: 10.3389/fpls.2021.729448 PMC8495254 34630474

[B36] Zhang J. Gu X. Yan W. Lou L. Xu X. Chen X. (2022). Characterization of differences in the composition and content of volatile compounds in cucumber fruit. Foods 11, 1101. doi: 10.3390/foods11081101 35454687 PMC9027996

[B37] Zhang A. Sun H. Wang Z. Sun W. Wang P. Wang X. (2010). Metabolomics: towards understanding traditional Chinese medicine. Planta Med. 76, 2026–2035. doi: 10.1055/s-0030-1250542 21058239

