# Supplementary material for: Non-targeted metabolomic analysis for the comparative evaluation of volatile organic compounds in 20 globally representative cucumber lines
Source: Front Plant Sci. 2022 Sep 29;13:1028735. doi: 10.3389/fpls.2022.1028735 (PMC9558236; doi:10.3389/fpls.2022.1028735)
Supplement: Supplementary file 1 [file DataSheet_1.docx]

Supplementary Material

**
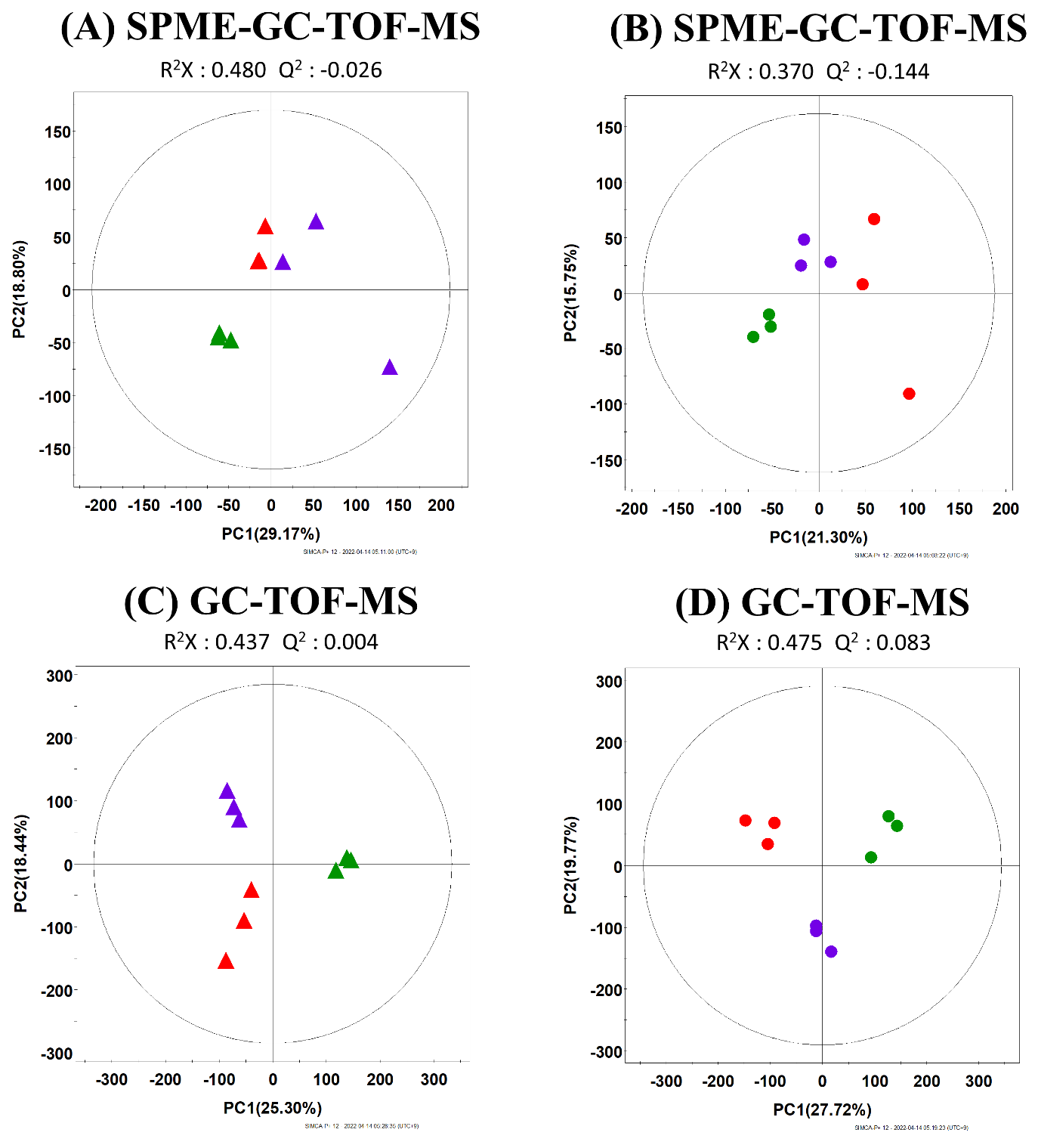
**

**Figure S1. PCA score plots of different three cucumbers based on SPME-GC-TOF-MS (A, B) and GC-TOF-MS (C, D) data set.** A and C are the results of analyzing different peel data. B and D are the results of analyzing different flesh data. Different sample symbolized as: Peel (▲: SJ24 (Korea group), ▲: SJ62 (Europe group), ▲: SJ109 (Thailand group)), flesh (●: SJ24 (Korea group), ●: SJ62 (Europe group), ●: SJ109 (Thailand group)).

**Table S1. Sample information of 20 cucumber breeding lines**

| **Line name** | **Group** | | **Fruit length (cm)** | | | **Fruit diameter (mm)** | | | **Fruit weight (g)** | | |
| --- | --- | --- | --- | --- | --- | --- | --- | --- | --- | --- | --- |
| **SJ01** | **Korea** | Korean solid green | 28.2 | ± | 2.1 | 45.2 | ± | 3.9 | 339.1 | ± | 67.8 |
| **SJ10** |  | Korean Semi-white | 26.5 | ± | 1.8 | 47.7 | ± | 5.2 | 349.8 | ± | 50.8 |
| **SJ24** |  | Korean Semi-white | 26.7 | ± | 3.4 | 45.1 | ± | 7.2 | 320.9 | ± | 108.1 |
| **SJ30** |  | Korea solid green | 29.4 | ± | 2.7 | 46.5 | ± | 5.1 | 354.6 | ± | 139.7 |
| **SJ80** | **Japan** | Japanese slicer | 24.0 | ± | 1.8 | 52.8 | ± | 0.9 | 410.1 | ± | 62.1 |
| **SJ67** | **China** | Chinese Long Green | 36.9 | ± | 5.1 | 75.2 | ± | 1.9 | 869.0 | ± | 35.0 |
| **SJ69** |  | Chinese Long Green | 19.9 | ± | 1.5 | 45.4 | ± | 1.1 | 252.1 | ± | 11.4 |
| **SJ37** | **Taiwan** | Taiwaness Slicer | 22.2 | ± | 4.6 | 67.5 | ± | 5.1 | 527.4 | ± | 54.9 |
| **SJ39** |  | Taiwaness Slicer | 14.2 | ± | 1.3 | 56.9 | ± | 0.2 | 273.1 | ± | 47.9 |
| **SJ86** | **Thailand** | Thai Slicer | 16.3 | ± | 1.0 | 38.3 | ± | 1.7 | 145.9 | ± | 34.2 |
| **SJ87** |  | Thai Slicer | 17.1 | ± | 0.5 | 47.5 | ± | 1.5 | 250.9 | ± | 14.9 |
| **SJ97** |  | Thai Slicer_White | 37.6 | ± | 4.7 | 36.3 | ± | 1.6 | 261.4 | ± | 38.5 |
| **SJ109** |  | Thai Slicer_White | 39.9 | ± | 1.5 | 39.9 | ± | 0.8 | 295.2 | ± | 23.7 |
| **SJ159** | **India** | India Wild type | 32.0 | ± | 2.8 | 46.3 | ± | 2.6 | 420.4 | ± | 4.6 |
| **SJ43** | **USA** | American Slicer | 17.0 | ± | 2.2 | 66.5 | ± | 6.4 | 410.4 | ± | 97.2 |
| **SJ50** |  | American Pickling | 19.8 | ± | 1.5 | 56.8 | ± | 2.3 | 380.5 | ± | 13.6 |
| **SJ262** |  | American Pickling | 18.8 | ± | 2.0 | 53.9 | ± | 5.8 | 355.6 | ± | 63.4 |
| **SJ62** | **Europe** | Beith Alpha | 81.9 | ± | 104.5 | 52.8 | ± | 1.1 | 310.1 | ± | 24.8 |
| **SJ65** |  | Beith Alpha | 7.9 | ± | 0.5 | 53.3 | ± | 5.5 | 129.0 | ± | 30.7 |
| **SJ46** | **-** | Parthenocarpic Slicer | 13.5 | ± | 0.7 | 54.0 | ± | 0.9 | 239.2 | ± | 19.0 |

**Table S2. Differential volatile organic compounds identified by HS-SPME-GC-TOF-MS in the peel of twenty cucumber breeding lines**

| **No.** | **Tentative**  **Identification^a^** | **HS-SPME-GC-TOF-MS** | | | | | |  |  |
| --- | --- | --- | --- | --- | --- | --- | --- | --- | --- |
|  |  | **Ret (min)^b^** | **Unique mass**  **(m/z)** | **MS fragment pattern(m/z)** | **CAS** | **Molecular Formula** | **Weight** | **ID** |  |
| ***Aldehydes*** | | | | | | | | |  |
| 1 | Propanal | 1.37 | 58 | 58,57,55,59,53,56,52,54,60 | 123-38-6 | C3H6O | 58 | Lib/MS^c^ |  |
| 2 | Pentanal | 2.11 | 58 | 58,57,55,53,56,50,51,71,67,59,86 | 110-62-3 | C5H10O | 86 | Lib/MS |  |
| 3 | Hexanal | 3.06 | 57 | 57,56,72,58,82,67,55,71,53,54,51 | 66-25-1 | C6H12O | 100 | Lib/MS |  |
| 4 | 2-Hexenal* | 4.89 | 98 | 55,69,83,57,56,70,98,53,80,54,97 | 505-57-7 | C6H10O | 98 | Lib/MS |  |
| 5 | 2-Octenal | 7.99 | 70 | 55,70,57,83,69,82,67,58,56,84,68 | 2548-87-0 | C8H14O | 126 | Lib/MS |  |
| 6 | 6-Nonenal | 8.01 | 93 | 55,54,81,67,70,93,79,69,83,56,53,68 | 2277-19-2 | C9H16O | 140 | Lib/MS |  |
| 7 | 2,4-Heptadienal* | 8.90 | 110 | 81,53,110,79,67,68,51,65,77,82,50 | 881395 | C7H10O | 110 | Lib/MS |  |
| 8 | Benzaldehyde | 9.25 | 105 | 77,105,106,95,51,81,50,79,53,124,78 | 100-52-7 | C7H6O | 106 | Lib/MS |  |
| 9 | 2,4-Nonadienal* | 11.59 | 138 | 81,67,53,82,54,55,83,79,68,138,95,65 | 5910-87-2 | C9H14O | 138 | Lib/MS |  |
| 10 | Tetradecanal* | 14.18 | 82 | 57,55,82,67,68,69,81,96,56,71,83,95 | 124-25-4 | C14H28O | 212 | Lib/MS |  |
| 11 | Hexadecanal* | 15.35 | 82 | 57,55,82,68,67,69,96,83,71,81,56,70 | 629-80-1 | C16H32O | 240 | Lib/MS |  |
| ***Alcohols*** | | | | | | | | |  |
| 12 | 3-Methyl-1  butanol | 4.80 | 55 | 55,70,57,56,69,53,71,54,58,50,51 | 123-51-3 | C5H12O | 88 | Lib/MS |  |
| 13 | 1-Hexanol* | 6.85 | 56 | 56,55,69,57,84,54,53,73,70,51,67,71 | 111-27-3 | C6H14O | 102 | Lib/MS |  |
| 14 | 1-Octen-3-ol | 8.28 | 57 | 57,72,55,58,85,68,81,67,53,99,82 | 3391-86-4 | C8H16O | 128 | Lib/MS |  |
| 15 | 3-Nonen-1-ol* | 11.33 | 68 | 55,68,67,81,69,54,82,57,95,56,53,70 | 10340-23-5 | C9H18O | 142 | Lib/MS |  |
| ***Etc.*** | | | | | | | | |  |
| 16 | 2-Ethylfuran | 1.92 | 81 | 81,96,53,67,95,82,65,51,50,52,97,63 | 3208-16-0 | C6H8O | 96 | Lib/MS |  |
| 17 | Octanoic acid  ethyl ester | 8.07 | 101 | 88,57,70,101,60,73,55,61,127,115,89 | 106-32-1 | C10H20O2 | 172 | Lib/MS |  |
| 18 | β-Ionone | 14.33 | 177 | 177,91,93,77,135,79,107,105,178,55 | 79-77-6 | C13H20O | 192 | Lib/MS |  |
| 19 | Octanoic acid | 15.57 | 101 | 60,73,55,101,87,84,61,85,69,56,57,115 | 124-07-2 | C8H16O2 | 144 | Lib/MS |  |
| ***N.I.*** | | | | | | | | |  |
| 20 | N.I.^d^ 1 | 2.64 | 70 | 70,69,71,55,50,57,56,68,53,51,52,72 | C4H6O | 70 | - | - |  |
| 21 | N.I. 2 | 11.50 | 68 | 68,57,69,55,97,84,53,56,51,79,81 | - | - | - | - |  |
| ^a^Identified volatile compounds based on variable importance projection(VIP) analysis with cut-off value of 0.7; ^b^ Retention time; ^c^MS, tentatively identified by mass spectra(comparison with Willy, Replib, Mainlib Library & HMDB, Pubchem database; ^d^N.I., Non-identified; **p*-value<0.05 | | | | | | | | |  |
|  |  |  |  |  |  |  |  |  |  |

**Table S3. Differential volatile organic compounds identified by HS-SPME-GC-TOF-MS in the flesh of twenty cucumber breeding lines**

| **No.** | **Tentative Identification^a^** | **HS-SPME-GC-TOF-MS** | | | | | |  |  |  |
| --- | --- | --- | --- | --- | --- | --- | --- | --- | --- | --- |
|  |  | **Ret (min)^b^** | **Unique mass**  **(m/z)** | **MS fragment pattern(m/z)** | **CAS** | **Molecular Formula** | **Weight** | **ID** | |  |
| ***Aldehydes*** | | | | | | | | |  |  |
| 1 | Propanal* | 1.37 | 58 | 58,57,55,59,53,56,52,54, | 123-38-6 | C3H6O | 58 | Lib/MS^c^ | |  |
| 2 | Pentanal* | 2.11 | 58 | 58,57,55,53,56,50,51,71 | 110-62-3 | C5H10O | 86 | Lib/MS | |  |
| 3 | Hexanal | 3.06 | 57 | 57,56,72,58,82,67,55,71 | 66-25-1 | C6H12O | 100 | Lib/MS | |  |
| 4 | 2-Hexenal* | 4.89 | 98 | 55,69,83,57,56,70,98,53 | 505-57-7 | C6H10O | 98 | Lib/MS | |  |
| 5 | 2-Octenal* | 7.99 | 70 | 55,70,57,83,69,82,67,58 | 2548-87-0 | C8H14O | 126 | Lib/MS | |  |
| 6 | 6-Nonenal | 8.01 | 93 | 55,54,81,67,70,93,79,69,83 | 2277-19-2 | C9H16O | 140 | Lib/MS | |  |
| 7 | 2,6-Nonadienal* | 10.12 | 70 | 70,69,67,53,68,55,81,94 | 557-48-2 | C9H14O | 138 | Lib/MS | |  |
| 8 | 2,4-Nonadienal* | 11.59 | 138 | 81,67,53,82,54,55,83,79,68,138 | 5910-87-2 | C9H14O | 138 | Lib/MS | |  |
| 9 | Tetradecanal* | 14.18 | 82 | 57,55,82,67,68,69,81,96,56 | 124-25-4 | C14H28O | 212 | Lib/MS | |  |
| 10 | Hexadecanal* | 15.35 | 82 | 57,55,82,68,67,69,96,83,71 | 629-80-1 | C16H32O | 240 | Lib/MS | |  |
| ***Alcohols*** | | | | | | | | |  |  |
| 11 | 1-Pentanol | 6.11 | 97 | 55,70,97,93,111,62,77,166 | 71-41-0 | C5H12O | 88 | Lib/MS | |  |
| 12 | 1-Hexanol* | 6.85 | 56 | 56,55,69,57,84,54,53,73,70 | 111-27-3 | C6H14O | 102 | Lib/MS | |  |
| 13 | 1-Nonanol* | 11.04 | 56 | 56,55,70,69,57,68,83,84,97 | 143-08-8 | C9H20O | 144 | Lib/MS | |  |
| 14 | 3-Nonen-1-ol* | 11.33 | 68 | 55,68,67,81,69,54,82,57,95 | 10340-23-5 | C9H18O | 142 | Lib/MS | |  |
| 15 | 6-Nonen-1-ol* | 11.73 | 67 | 67,55,68,82,95,81,54,69,57 | 35854-86-5 | C9H18O | 142 | Lib/MS | |  |
| 16 | 3,6-Nonadien-1-ol* | 12.12 | 93 | 67,79,93,55,91,77,81,68,53 | 56805-23-3 | C9H16O | 140 | Lib/MS | |  |
| ***Etc.*** |  |  |  |  |  |  |  |  | |  |
| 17 | 2-Ethylfuran* | 1.92 | 81 | 81,96,53,67,95,82,65,51,50 | 3208-16-0 | C6H8O | 96 | Lib/MS | |  |
| 18 | β-Ionone* | 14.33 | 177 | 177,91,93,77,135,79,107,105 | 79-77-6 | C13H20O | 192 | Lib/MS | |  |
| 19 | Nonanoic acid* | 16.36 | 115 | 60,73,57,55,115,61,129,87 | 112-05-0 | C9H18O2 | 158 | Lib/MS | |  |
| ^a^Identified volatile compounds based on variable importance projection(VIP) analysis with cut-off value of 0.7; ^b^ Retention time; ^c^MS, tentatively identified by mass spectra(comparison with Willy, Replib, Mainlib Library & HMDB, Pubchem database; ^d^N.I., Non-identified; **p*-value<0.05 | | | | | | | | |  |  |
|  |  |  |  |  |  |  |  |  |  | |

**Table S4. Relative abundance value of discriminant VOCs in the peel of twenty cucumber lines based on HS-SPME-GC-TOF-MS data.**

**(A) Mean**

|  | | ***Aldehydes*** | | | | | | | | | | |
| --- | --- | --- | --- | --- | --- | --- | --- | --- | --- | --- | --- | --- |
|  |  | **Propanal** | **Hexadecanal*** | **Tetradecanal*** | **2-Hexenal*** | **Hexanal** | **6-Nonenal** | **Benzaldehyde** | **Pentanal** | **2,4-Nonadienal*** | **2,4-Heptadienal*** | **2-Octenal** |
| **Korea** | **SJ01** | 3542743.7 | 1286308.3 | 125854.7 | 38005.3 | 1593349.0 | 389678.0 | 1196863.3 | 3007540.3 | 4481093.0 | 453517.7 | 3144252.0 |
|  | **SJ10** | 2406790.0 | 537361.0 | - | 263209.3 | 3271824.7 | 725781.0 | 2048745.7 | 3101958.0 | 2334124.3 | 183336.3 | 25061.3 |
|  | **SJ24** | 4633059.0 | 4007720.0 | 330152.0 | 131204.0 | 1970489.3 | 389180.0 | 1505778.7 | 2420957.0 | 1869106.7 | 407599.0 | 85509.3 |
|  | **SJ30** | 5958683.7 | 456668.0 | - | 265102.3 | 2719879.3 | 304043.3 | 1404359.3 | 2333507.7 | 1995623.3 | 373205.7 | - |
| **Japan** | **SJ80** | 3428637.0 | 1890167.7 | - | 92916.7 | 1899960.0 | 290064.0 | 1788755.3 | 3190937.0 | 5984080.0 | 359175.7 | 166099.0 |
| **China** | **SJ67** | 5563390.3 | 9510684.7 | 699770.0 | 98067.3 | 2166554.7 | 517968.0 | 2245056.7 | 3679020.3 | 6899915.3 | 306320.3 | 287153.3 |
|  | **SJ69** | 4943875.7 | 1856304.3 | 219712.3 | 106425.0 | 2109989.0 | 315267.3 | 1410843.7 | 4804860.3 | 6637048.7 | 330015.3 | 160381.0 |
| **Taiwan** | **SJ37** | 3311786.0 | 971239.3 | 106405.7 | 247204.7 | 2567317.3 | 342357.7 | 1275516.3 | 3435236.0 | 4154797.7 | 421968.3 | 3963670.0 |
|  | **SJ39** | 3085938.3 | 158025.7 | - | 202813.3 | 2505356.7 | 484799.7 | 856183.3 | 3974783.0 | 5988861.7 | 470582.3 | - |
| **Thailand** | **SJ86** | 4237459.0 | 1697209.3 | 181426.8 | 140826.3 | 5381111.0 | 1730850.7 | 2567335.7 | 14207341.0 | 11231436.7 | 264110.3 | 108470.0 |
|  | **SJ87** | 4996962.0 | 2215792.0 | 280666.3 | 108124.3 | 5733519.7 | 1768889.3 | 2775008.0 | 7664479.3 | 10581773.7 | 299213.0 | 24912.7 |
|  | **SJ97** | 4802248.3 | 1087300.7 | 35802.0 | 169065.3 | 5643434.0 | 1487322.7 | 1735788.7 | 4542934.3 | 8143898.3 | 266765.0 | 5112.3 |
|  | **SJ109** | 3660513.3 | 3070676.3 | 274528.7 | 314635.7 | 4083910.7 | 1172186.3 | 2205851.7 | 5264102.0 | 3604450.3 | 97115.3 | - |
| **India** | **SJ159** | 5247333.0 | 32605625.0 | 2760858.0 | 238228.7 | 845091.7 | 63825.7 | 1465594.0 | - | 429951.3 | 202396.0 | 48644.7 |
| **USA** | **SJ43** | 6743593.7 | 1326307.0 | - | 96579.7 | 2059818.7 | 414864.7 | 1749512.0 | 2631507.0 | 2290109.3 | 588495.0 | - |
|  | **SJ50** | 9709938.0 | 205227.3 | - | 277233.0 | 2762576.3 | 511689.0 | 1203041.0 | 2445797.7 | 2146813.3 | 529974.7 | 112739.3 |
|  | **SJ262** | 6038997.3 | 208018.7 | - | 194331.7 | 4580098.7 | 819261.3 | 2204534.3 | 4628030.0 | 3862025.7 | 18014.0 | 60723.3 |
| **Europe** | **SJ62** | 8545655.0 | 1313304.0 | 123872.0 | 197185.3 | 773483.3 | 282687.7 | 1136814.3 | 1046920.3 | 978861.3 | 298454.0 | 151727.0 |
|  | **SJ65** | 3385777.0 | 2707321.7 | 311577.3 | 129450.3 | 710383.0 | 234248.3 | 1576443.3 | 2206740.0 | 984182.0 | 410081.3 | 1279396.3 |
| **Etc.** | **SJ46** | 4330586.7 | 987931.3 | - | 231483.3 | 3224310.7 | 588307.7 | 2351319.0 | 4292380.3 | 4449915.0 | 491724.0 | - |

**Cont. (Table S4. (A) Mean)**

|  | | ***Alcohols*** | | | | ***Etc.*** | | | | ***N.I.*** | |
| --- | --- | --- | --- | --- | --- | --- | --- | --- | --- | --- | --- |
|  |  | **1-Octen-3-ol** | **3-Methyl- 1-butanol** | **1-Hexanol*** | **3-Nonen-1-ol*** | **Octanoic acid  ethyl ester** | **Octanoic acid** | **β-Ionone** | **2-Ethylfuran** | **N.I. 1** | **N.I. 2** |
| **Korea** | **SJ01** | 8258358.3 | 300666.0 | 775277.0 | 26698780.0 | 13004962.3 | 1196469.0 | 5886002.7 | 684844.7 | 1731364.7 | 1779841.0 |
|  | **SJ10** | 4374359.7 | 1550517.3 | 1782786.0 | - | 28959.0 | 302484.0 | 1278815.7 | 392391.7 | 456361.3 | 490615.3 |
|  | **SJ24** | 4657156.3 | 824529.3 | 2841401.0 | 5514211.3 | 149216.7 | 1146246.3 | 2797228.0 | 458105.0 | 900682.7 | 677796.7 |
|  | **SJ30** | 4558570.7 | 1684027.7 | 96663.0 | 6989491.3 | 1453557.7 | 1118857.3 | 2956163.3 | 502669.0 | - | 823238.7 |
| **Japan** | **SJ80** | 6593499.7 | 591023.0 | 966024.3 | 11687266.3 | 1196793.3 | 1819207.0 | 5976638.0 | 1000575.3 | 2243915.0 | 900171.7 |
| **China** | **SJ67** | 8362703.0 | 620955.7 | 1180651.0 | - | 927172.3 | 277225.7 | 6174389.7 | 1618102.7 | 417376.7 | 337804.0 |
|  | **SJ69** | 4140258.7 | 687660.0 | 2484630.7 | - | 3167264.7 | 2523668.0 | 5842960.0 | 1342852.3 | 1349875.3 | 786069.0 |
| **Taiwan** | **SJ37** | 6020632.7 | 1553802.0 | 525388.0 | 5028041.3 | 2410220.0 | 1489607.0 | 2917379.7 | 763899.3 | - | 655853.0 |
|  | **SJ39** | 5884362.0 | 1307890.0 | 773924.0 | 7005867.7 | 2117993.3 | 905174.0 | 2531166.3 | 1324977.7 | - | 720635.7 |
| **Thailand** | **SJ86** | 15373111.0 | 875844.7 | 502691.3 | 1938846.3 | 1304782.3 | 536737.7 | 753412.7 | 729029.7 | 262582.7 | 207852.0 |
|  | **SJ87** | 12197943.0 | 692738.0 | 8006996.0 | 1345155.7 | 46235.0 | 151231.0 | 1145999.3 | 1391997.3 | 165283.3 | 120200.3 |
|  | **SJ97** | 12797637.0 | 1063214.0 | 526836.3 | 1019913.3 | 395560.0 | 137944.3 | 68886.7 | 1248172.7 | - | 103967.0 |
|  | **SJ109** | 8615619.3 | 2020229.3 | 6360059.7 | 369635.8 | 946996.0 | 606487.3 | 98634.0 | 742951.3 | 916670.7 | 41757.3 |
| **India** | **SJ159** | 1452131.0 | 1597000.3 | 10174059.3 | 1058386.0 | 34597.0 | 350462.3 | 2490137.7 | 295736.3 | 494893.3 | 137166.7 |
| **USA** | **SJ43** | 5938353.3 | 618535.3 | 1400366.0 | 15225660.3 | 152816.0 | 719283.7 | 4510107.7 | 1549075.3 | 57790.7 | 1916220.0 |
|  | **SJ50** | 4866562.0 | 1763754.3 | 3307476.0 | 13114182.7 | 245329.3 | 130704.3 | 2360919.7 | 1675506.0 | 259902.3 | 1897951.0 |
|  | **SJ262** | 6968180.3 | 1270470.0 | 17134835.3 | 2183933.0 | 755385.3 | 658710.0 | 1130396.0 | 1369949.0 | 392355.3 | 320217.7 |
| **Europe** | **SJ62** | 2556384.7 | 1275380.7 | 3118015.3 | 8989068.7 | 81853.3 | 759248.3 | 4373541.7 | 631444.3 | - | 1813959.0 |
|  | **SJ65** | 3130144.0 | 858381.7 | 4372062.0 | - | 705498.7 | 1932532.3 | 3328731.7 | 606294.7 | - | 1698725.7 |
| **Etc.** | **SJ46** | 7179731.0 | 1455538.7 | 3263332.0 | 2741485.3 | 600182.0 | 760380.0 | 3375401.0 | 1316009.3 | 241335.0 | 749702.3 |

Relative abundance values of significantly discriminant metabolites (VIP > 0.7) in twenty cucumber lines based on PLS-DA model (Figure 2B). HS-SPME-GC-TOF-MS, headspace-solid phase microextraction gas chromatography time-of-flight mass spectrometry; VIP, variable importance projection; PLS-DA, partial least squares-discriminant analysis. **p*-value < 0.05, ^d^N.I., Non-identified; -, non-detected compounds

**(B) Standard deviation**

|  | | ***Aldehydes*** | | | | | | | | | | | |
| --- | --- | --- | --- | --- | --- | --- | --- | --- | --- | --- | --- | --- | --- |
|  |  | **Propanal** | **Hexadecanal*** | **Tetradecanal*** | **2-Hexenal*** | **Hexanal** | **6-Nonenal** | **Benzaldehyde** | **Pentanal** | **2,4-Nonadienal*** | **2,4-Heptadienal*** | **2-Octenal** |  |
| **Korea** | **SJ01** | 913384.1 | 796692.4 | 138463.1 | 8218.6 | 510891.2 | 80613.0 | 152529.8 | 644956.7 | 1265957.1 | 68688.5 | 4810527.1 |  |
|  | **SJ10** | 778913.2 | 449473.4 | - | 171281.4 | 1495162.3 | 313832.1 | 608974.3 | 1048258.6 | 1524610.0 | 88174.5 | 43407.5 |  |
|  | **SJ24** | 424835.9 | 2998874.6 | 215142.0 | 40411.3 | 603013.2 | 137437.5 | 398549.0 | 479968.5 | 444403.9 | 87163.4 | 35454.2 |  |
|  | **SJ30** | 1975941.4 | 117711.8 | - | 69457.1 | 723090.4 | 92596.9 | 607196.5 | 354534.3 | 866815.2 | 131695.8 | - |  |
| **Japan** | **SJ80** | 753524.6 | 2235823.9 | - | 25889.7 | 687128.5 | 129242.9 | 1742703.3 | 1076382.2 | 2243556.7 | 57128.7 | 55160.6 |  |
| **China** | **SJ67** | 835408.5 | 5327282.6 | 486869.5 | 23997.9 | 591970.5 | 223530.4 | 667074.0 | 740503.4 | 2975595.8 | 77010.2 | 137528.8 |  |
|  | **SJ69** | 681241.5 | 602930.0 | 70422.2 | 9565.6 | 47421.2 | 30811.7 | 688943.2 | 563269.4 | 1008805.9 | 52655.6 | 71144.0 |  |
| **Taiwan** | **SJ37** | 736522.9 | 413054.0 | 83283.6 | 140579.7 | 759717.7 | 117224.9 | 451941.6 | 906728.0 | 2819159.9 | 171278.4 | 4064494.9 |  |
|  | **SJ39** | 464633.8 | 25518.8 | - | 99288.8 | 858003.9 | 260056.2 | 55184.4 | 1551720.3 | 3683843.6 | 135267.8 | - |  |
| **Thailand** | **SJ86** | 739901.8 | 119648.2 | 172222.9 | 19165.5 | 602218.0 | 221216.5 | 46698.7 | 3776247.5 | 1191777.3 | 199851.9 | 70105.7 |  |
|  | **SJ87** | 347117.0 | 699723.1 | 156519.9 | 37584.6 | 1145283.6 | 267309.9 | 229270.2 | 3364940.0 | 3930989.6 | 34989.9 | 7736.5 |  |
|  | **SJ97** | 2820877.3 | 680757.5 | 32102.3 | 76656.5 | 688416.7 | 349643.7 | 165997.1 | 487356.6 | 2009739.3 | 133578.1 | 8854.8 |  |
|  | **SJ109** | 1019626.0 | 1249834.2 | 73028.4 | 140477.7 | 1260928.6 | 67010.9 | 504448.5 | 2132495.1 | 935970.6 | 72749.0 | - |  |
| **India** | **SJ159** | 5726467.7 | 10561158.1 | 812585.7 | 103074.8 | 159736.0 | 10143.3 | 579252.3 | - | 14093.5 | 32112.5 | 46816.9 |  |
| **USA** | **SJ43** | 893465.4 | 543400.7 | - | 2811.1 | 623980.7 | 64926.9 | 443780.1 | 540595.0 | 366146.3 | 86597.1 | - |  |
|  | **SJ50** | 1896061.2 | 35256.4 | - | 28356.8 | 1049760.7 | 154317.2 | 147298.5 | 653502.4 | 1320765.2 | 109594.6 | 132528.1 |  |
|  | **SJ262** | 2020463.2 | 53316.6 | - | 41832.9 | 225583.8 | 125998.2 | 386714.5 | 794527.4 | 857468.2 | 14058.3 | 38668.5 |  |
| **Europe** | **SJ62** | 4112890.1 | 333389.4 | 19917.2 | 71408.8 | 258703.3 | 63919.2 | 430011.3 | 232497.1 | 130689.8 | 90987.5 | 62369.8 |  |
|  | **SJ65** | 948132.7 | 326294.9 | 151436.0 | 41111.3 | 225787.5 | 68154.4 | 246955.8 | 262160.7 | 162407.4 | 156471.4 | 845269.8 |  |
| **Etc.** | **SJ46** | 562740.1 | 287447.7 | - | 71784.5 | 655767.7 | 95292.1 | 808061.1 | 984807.1 | 1321367.4 | 84022.5 | - |  |

**Cont. (Table S4. (B) Standard deviation)**

|  | | ***Alcohols*** | | | | ***Etc.*** | | | | ***N.I.*** | |
| --- | --- | --- | --- | --- | --- | --- | --- | --- | --- | --- | --- |
|  |  | **1-Octen-3-ol** | **3-Methyl- 1-butanol** | **1-Hexanol*** | **3-Nonen-1-ol*** | **Octanoic acid ethyl ester** | **Octanoic acid** | **β-Ionone** | **2-Ethylfuran** | **N.I. 1** | **N.I. 2** |
| **Korea** | **SJ01** | 1135600.2 | 133020.4 | 77986.9 | 4172670.9 | 7039788.1 | 834268.4 | 1722210.0 | 330986.7 | 931036.6 | 385163.8 |
|  | **SJ10** | 1840919.0 | 1070319.3 | 417539.2 | - | 50158.5 | 134771.3 | 780520.2 | 157423.7 | 393645.1 | 485460.4 |
|  | **SJ24** | 749188.8 | 269776.7 | 1343052.5 | 904102.9 | 158287.1 | 617065.6 | 1416202.0 | 87997.4 | 872591.8 | 12883.7 |
|  | **SJ30** | 3825177.4 | 483666.5 | 74649.5 | 9283051.2 | 79874.6 | 773584.9 | 396073.6 | 174503.6 | - | 422395.9 |
| **Japan** | **SJ80** | 2687038.5 | 163757.5 | 1628753.6 | 5011227.7 | 789062.7 | 1153783.8 | 640498.5 | 588961.5 | 2683184.7 | 336387.0 |
| **China** | **SJ67** | 998357.8 | 164150.6 | 65344.6 | - | 160467.6 | 237065.1 | 666574.5 | 229549.7 | 204717.2 | 31706.3 |
|  | **SJ69** | 2849814.5 | 369100.0 | 941311.6 | - | 1265977.8 | 391113.9 | 1072218.0 | 321225.8 | 310016.4 | 254780.2 |
| **Taiwan** | **SJ37** | 3016152.2 | 844453.6 | 467395.9 | 1941242.7 | 2057404.4 | 1146333.1 | 236252.2 | 299986.6 | - | 159606.1 |
|  | **SJ39** | 2655121.9 | 607388.2 | 223847.8 | 2110549.8 | 1531746.9 | 479489.9 | 679117.6 | 674753.9 | - | 219398.6 |
| **Thailand** | **SJ86** | 1471337.7 | 127298.7 | 372774.4 | 157040.8 | 988903.3 | 309901.0 | 322999.8 | 228605.4 | 108989.0 | 31323.9 |
|  | **SJ87** | 3313941.5 | 222847.0 | 10696769.7 | 85948.6 | 33773.9 | 102427.7 | 612723.9 | 427470.3 | 51617.6 | 50374.4 |
|  | **SJ97** | 4147858.4 | 484685.3 | 829716.9 | 215346.0 | 378750.2 | 39144.0 | 2503.4 | 551322.4 | - | 53241.5 |
|  | **SJ109** | 515408.1 | 878824.3 | 2516437.7 | 312405.7 | 758866.7 | 440216.9 | 56691.7 | 220367.6 | 248371.6 | 9987.2 |
| **India** | **SJ159** | 90224.5 | 732904.7 | 4405527.1 | 71644.5 | 14599.1 | 342567.2 | 453333.6 | 141180.5 | 313690.3 | 30473.9 |
| **USA** | **SJ43** | 1811496.1 | 13937.7 | 1185831.4 | 4985313.7 | 97120.7 | 531869.8 | 20700.7 | 997782.1 | 24219.4 | 325415.7 |
|  | **SJ50** | 1433291.7 | 141629.3 | 2253754.8 | 4323746.7 | 184094.7 | 10356.1 | 328036.0 | 38189.3 | 186425.6 | 981764.4 |
|  | **SJ262** | 1575338.1 | 288339.0 | 16736626.8 | 942866.9 | 10358.3 | 370307.0 | 212375.9 | 593171.3 | 167162.7 | 180005.1 |
| **Europe** | **SJ62** | 546923.7 | 476026.9 | 268716.9 | 1808850.2 | 92771.4 | 824606.8 | 573956.7 | 162739.3 | - | 283783.7 |
|  | **SJ65** | 514832.4 | 261656.1 | 1343815.0 | - | 525828.2 | 998014.9 | 185652.4 | 301499.7 | - | 951612.1 |
| **Etc.** | **SJ46** | 1354430.4 | 461953.3 | 1381534.4 | 3139006.8 | 870363.9 | 651181.2 | 165280.6 | 517650.8 | 160275.7 | 249834.4 |

Relative abundance values of significantly discriminant metabolites (VIP > 0.7) in twenty cucumber lines based on PLS-DA model (Figure 2B). HS-SPME-GC-TOF-MS, headspace-solid phase microextraction gas chromatography time-of-flight mass spectrometry; VIP, variable importance projection; PLS-DA, partial least squares-discriminant analysis. **p*-value < 0.05, ^d^N.I., Non-identified; -, non-detected compounds

**Table S5. Relative abundance value of discriminant VOCs in the flesh of twenty cucumber lines based on HS-SPME-GC-TOF-MS data.**

**(A) Mean**

|  | | ***Aldehydes*** | | | | | | | | | |
| --- | --- | --- | --- | --- | --- | --- | --- | --- | --- | --- | --- |
|  |  | **2,6-Nonadienal*** | **Propanal*** | **Pentanal*** | **2,4-Nonadienal*** | **2-Octenal*** | **Tetradecanal*** | **Hexadecanal*** | **Hexanal** | **2-Hexenal*** | **6-Nonenal** |
| **Korea** | **SJ01** | 2078048.7 | 444329.3 | 962986.0 | 9312.3 | 4344403.3 | 345218.0 | 1015231.0 | 391504.0 | 14649.7 | 394860.7 |
|  | **SJ10** | 1509537.7 | 357020.3 | 559383.3 | 3466.7 | 1546577.0 | 132224.0 | 909702.0 | 540961.0 | 40982.0 | 1155862.0 |
|  | **SJ24** | 1548915.0 | 253128.0 | 831604.7 | 6806.3 | 2092299.3 | 267816.0 | 1265928.3 | 269980.7 | 16764.0 | 3074173.0 |
|  | **SJ30** | 2929775.3 | 586015.3 | 1160949.3 | 5787.3 | 1574280.0 | 301274.7 | 1166699.3 | 311638.7 | 25383.7 | 52593.3 |
| **Japan** | **SJ80** | 2713119.0 | 496852.3 | 1072847.3 | 5490.3 | 2752014.3 | 307243.3 | 1096925.3 | 321832.0 | 27380.0 | 1195424.0 |
| **China** | **SJ67** | 2369733.3 | 652633.3 | 1523648.0 | 9111.0 | 5963514.7 | 566182.3 | 1185899.0 | 422461.7 | 8419.3 | 21672.3 |
|  | **SJ69** | 2634226.7 | 732650.0 | 1931092.0 | 15330.0 | 8473151.3 | 216811.7 | 899277.7 | 656103.3 | 29743.3 | 196543.0 |
| **Taiwan** | **SJ37** | 2525343.3 | 310073.7 | 744206.7 | 6431.0 | 2370296.0 | 215239.0 | 924181.0 | 407962.0 | 44417.7 | 836585.3 |
|  | **SJ39** | 2961603.7 | 360259.7 | 1289269.7 | 17798.7 | 7430938.0 | 225646.3 | 882457.3 | 714244.0 | 14786.3 | 190448.3 |
| **Thailand** | **SJ86** | 3353525.7 | 362942.3 | 1121148.3 | 10959.7 | 3612395.3 | 233107.0 | 1202019.0 | 465126.0 | 34054.7 | 623710.3 |
|  | **SJ87** | 3006441.0 | 611914.7 | 1842053.7 | 12298.3 | 6428593.0 | 477551.0 | 1163874.7 | 463616.0 | 28255.0 | 820892.7 |
|  | **SJ97** | 3546918.3 | 586987.0 | 1107766.7 | 9353.3 | 3462234.0 | 277500.0 | 1189308.3 | 503054.7 | 37573.7 | 363398.3 |
|  | **SJ109** | 3038440.3 | 808802.0 | 1540779.3 | 12215.3 | 3805025.3 | 300116.0 | 1031785.3 | 610043.7 | 41369.0 | 344244.3 |
| **India** | **SJ159** | 2550119.0 | 711509.3 | 1102822.7 | 2603.3 | 574126.7 | 331693.3 | 1225777.0 | 133020.3 | 26413.7 | 1290076.3 |
| **USA** | **SJ43** | 2719800.0 | 700078.3 | 1166761.0 | 5146.3 | 1871400.0 | 282257.0 | 994864.0 | 296909.7 | 49871.3 | 897054.3 |
|  | **SJ50** | 3459368.3 | 562746.7 | 1190316.7 | 13618.3 | 3868846.0 | 114437.0 | 788165.3 | 515133.7 | 36106.7 | 749911.7 |
|  | **SJ262** | 2946455.7 | 377831.3 | 953012.0 | 14321.3 | 2860233.7 | 135899.7 | 936493.0 | 389246.7 | 20381.0 | 2632392.3 |
| **Europe** | **SJ62** | 3638353.3 | 1030320.0 | 1290464.7 | 12698.7 | 6311921.0 | 341278.0 | 1158896.0 | 658350.3 | 23755.3 | 125676.7 |
|  | **SJ65** | 1891600.7 | 169945.3 | 755395.3 | 7500.0 | 3487109.3 | 284607.3 | 1183453.3 | 536593.7 | 26895.3 | 128538.7 |
| **Etc.** | **SJ46** | 2531736.7 | 516857.3 | 1923349.7 | 14846.7 | 1871400.0 | 399374.0 | 958134.3 | 668117.0 | 32464.3 | 729073.0 |

**Cont. (Table S5. (A) Mean)**

|  | | ***Alcohols*** | | | | | | ***Etc.*** | | | |
| --- | --- | --- | --- | --- | --- | --- | --- | --- | --- | --- | --- |
|  |  | **3-Nonen-1-ol*** | **3,6-Nonadien-1-ol*** | **6-Nonen-1-ol*** | **1-Pentanol** | **1-Hexanol*** | **1-Nonanol*** | | **Nonanoic acid*** | **2-Ethylfuran*** | **β-Ionone*** |
| **Korea** | **SJ01** | 146605.7 | 50878.3 | 144872.7 | 989407.3 | 3247955.0 | 274135.3 | | 249256.3 | 29289.0 | 372573.0 |
|  | **SJ10** | 503666.7 | 113030.3 | 628672.7 | 597198.7 | 28702913.7 | 1040240.3 | | 830129.3 | 16777.7 | 179463.3 |
|  | **SJ24** | 161952.0 | 37338.7 | 142673.7 | 538023.3 | 3576571.7 | 264380.7 | | 185732.3 | 8634.0 | 411175.7 |
|  | **SJ30** | 106887.0 | 21950.7 | 56771.7 | 440099.7 | 3822333.3 | 545739.3 | | 273942.3 | 18117.0 | 315620.0 |
| **Japan** | **SJ80** | 85758.3 | 65617.3 | 126756.3 | 231487.0 | 7581699.0 | 198789.7 | | 5585424.7 | 37013.7 | 483400.0 |
| **China** | **SJ67** | 40641.3 | 30831.7 | 45210.7 | 572763.7 | 3644768.7 | 26869.0 | | 5461827.0 | 89853.7 | 441135.3 |
|  | **SJ69** | 71165.0 | 11592.0 | 27805.3 | 622054.3 | 910106.7 | 54110.7 | | 8394536.7 | 52359.7 | 536907.7 |
| **Taiwan** | **SJ37** | 127208.0 | 69567.0 | 203981.7 | 634186.3 | 16788470.7 | 1147438.3 | | 954759.7 | 18272.3 | 201380.0 |
|  | **SJ39** | 103375.3 | 6136.3 | 104084.0 | 1175051.0 | 2189635.3 | 603294.7 | | 233465.3 | 22498.0 | 158168.0 |
| **Thailand** | **SJ86** | 68145.7 | 28029.7 | 54203.7 | 878330.0 | 4057810.3 | 387288.0 | | 5677838.7 | 32963.3 | 190203.3 |
|  | **SJ87** | 29019.3 | 20361.7 | 47498.3 | 1299240.3 | 1826360.0 | 129417.7 | | 4160625.0 | 50389.3 | 499355.0 |
|  | **SJ97** | 35368.0 | 21763.3 | 60643.7 | 706094.3 | 2990984.3 | 270464.7 | | 304527.0 | 47890.3 | 169608.0 |
|  | **SJ109** | 65984.7 | 9485.7 | 60211.0 | 1196189.7 | 1435268.7 | 194329.3 | | 327550.3 | 72114.0 | 367642.0 |
| **India** | **SJ159** | 49636.7 | 6723.7 | 195250.0 | 545933.3 | 9902868.0 | 329250.0 | | 116665.7 | 15406.0 | 887107.3 |
| **USA** | **SJ43** | 32537.7 | 9455.7 | 42757.3 | 597197.0 | 1211959.7 | 140347.0 | | 124148.7 | 17993.3 | 765393.3 |
|  | **SJ50** | 95403.7 | 24150.7 | 213382.3 | 752584.3 | 4538524.3 | 784404.0 | | 3587566.3 | 29206.0 | 396688.0 |
|  | **SJ262** | 59376.7 | 14392.0 | 82894.0 | 1076519.0 | 20273014.7 | 835487.0 | | 492890.3 | 45548.3 | 314400.7 |
| **Europe** | **SJ62** | 52805.7 | 39146.7 | 75635.3 | 1060825.3 | 1984532.7 | 151348.7 | | 5224842.7 | 68920.0 | 524632.3 |
|  | **SJ65** | 83688.7 | 46680.3 | 89991.7 | 1475690.0 | 39921400.0 | 165001.3 | | 3523445.7 | 59911.0 | 579685.0 |
| **Etc.** | **SJ46** | 115092.0 | 47732.3 | 183280.0 | 597197.0 | 1244524.3 | 809571.7 | | 125922.0 | 40016.3 | 233550.7 |

Relative abundance values of significantly discriminant metabolites (VIP > 0.7) in twenty cucumber lines based on PLS-DA model (Figure 2E). HS-SPME-GC-TOF-MS, headspace-solid phase microextraction gas chromatography time-of-flight mass spectrometry; VIP, variable importance projection; PLS-DA, partial least squares-discriminant analysis. **p*-value < 0.05, ^d^N.I., Non-identified; -, non-detected compounds

**(B) Standard deviation**

|  | | ***Aldehydes*** | | | | | | | | | |
| --- | --- | --- | --- | --- | --- | --- | --- | --- | --- | --- | --- |
|  |  | **2,6-Nonadienal*** | **Propanal*** | **Pentanal*** | **2,4-Nonadienal*** | **2-Octenal*** | **Tetradecanal*** | **Hexadecanal*** | **Hexanal** | **2-Hexenal*** | **6-Nonenal** |
| **Korea** | **SJ01** | 793558.3 | 141576.6 | 370727.8 | 1306.8 | 1731266.5 | 61637.6 | 129055.4 | 217794.5 | 5705.9 | 341458.6 |
|  | **SJ10** | 148674.1 | 56519.7 | 97428.7 | 954.8 | 234216.2 | 21522.9 | 186586.0 | 109096.8 | 17213.9 | 855054.0 |
|  | **SJ24** | 581263.0 | 81937.2 | 21253.8 | 3647.4 | 915257.8 | 50408.6 | 131358.9 | 129397.4 | 8438.5 | 4044930.7 |
|  | **SJ30** | 222166.0 | 85475.2 | 170357.9 | 1057.1 | 263235.5 | 63962.1 | 98668.0 | 29438.6 | 3605.4 | 16074.0 |
| **Japan** | **SJ80** | 453262.7 | 242872.2 | 184127.4 | 2901.8 | 1718418.7 | 68091.3 | 2254.0 | 188401.0 | 23605.1 | 1459094.9 |
| **China** | **SJ67** | 349199.0 | 256232.1 | 245372.5 | 1999.8 | 2951013.1 | 64101.0 | 62751.2 | 157742.7 | 2481.9 | 7597.1 |
|  | **SJ69** | 943921.1 | 55662.8 | 247858.1 | 864.9 | 2256901.0 | 22461.0 | 90382.9 | 72821.0 | 13695.0 | 119225.0 |
| **Taiwan** | **SJ37** | 126808.0 | 82719.2 | 131542.7 | 4122.1 | 1557530.6 | 48958.0 | 141349.0 | 50156.0 | 19698.7 | 455383.1 |
|  | **SJ39** | 640407.4 | 93470.0 | 338257.5 | 4211.4 | 2625972.8 | 56538.5 | 15816.7 | 175989.0 | 2480.3 | 19400.8 |
| **Thailand** | **SJ86** | 221470.0 | 200199.6 | 333617.5 | 8793.0 | 3828950.8 | 56294.1 | 55929.3 | 446882.7 | 16803.0 | 678629.7 |
|  | **SJ87** | 765730.2 | 117043.8 | 694006.6 | 2539.9 | 4829582.0 | 125620.5 | 275454.3 | 212516.5 | 6834.0 | 885975.1 |
|  | **SJ97** | 252887.9 | 305944.4 | 246972.7 | 6780.6 | 2810981.8 | 137653.6 | 105855.1 | 309437.8 | 21626.2 | 223031.5 |
|  | **SJ109** | 805699.2 | 292288.3 | 866475.7 | 8943.7 | 3619122.4 | 29453.5 | 137748.4 | 536978.3 | 15124.7 | 129869.2 |
| **India** | **SJ159** | 410197.5 | 85471.3 | 198306.1 | 2438.3 | 361354.3 | 43591.4 | 173177.0 | 122711.4 | 10597.7 | 187538.9 |
| **USA** | **SJ43** | 1044629.4 | 163654.4 | 343620.5 | 1651.1 | 906608.4 | 52389.9 | 36629.0 | 110471.7 | 18047.8 | 680060.1 |
|  | **SJ50** | 541389.5 | 229420.2 | 478344.4 | 8165.4 | 2811863.8 | 42344.9 | 308916.4 | 244160.6 | 9113.4 | 661784.0 |
|  | **SJ262** | 162317.8 | 84987.5 | 166617.2 | 3970.3 | 41809.1 | 34751.5 | 218898.7 | 87170.4 | 1843.3 | 517023.0 |
| **Europe** | **SJ62** | 435149.2 | 238938.6 | 247866.2 | 3367.6 | 4308921.9 | 59716.4 | 17792.2 | 257367.8 | 2906.8 | 40175.8 |
|  | **SJ65** | 1033435.7 | 73458.4 | 294715.1 | 4371.6 | 2666978.2 | 164350.3 | 202695.6 | 431798.2 | 15489.2 | 60479.1 |
| **Etc.** | **SJ46** | 462799.4 | 53027.8 | 601130.0 | 1604.1 | 906608.4 | 98059.3 | 98668.2 | 139213.2 | 8891.1 | 484236.5 |

**Cont. (Table S5 (B) Standard deviation)**

|  | | ***Alcohols*** | | | | | | ***Etc.*** | | |
| --- | --- | --- | --- | --- | --- | --- | --- | --- | --- | --- |
|  |  | **3-Nonen-1-ol*** | **3,6-Nonadien-1-ol*** | **6-Nonen-1-ol*** | **1-Pentanol** | **1-Hexanol*** | **1-Nonanol*** | **Nonanoic acid*** | **2-Ethylfuran*** | **β-Ionone*** |
| **Korea** | **SJ01** | 22925.1 | 12232.9 | 97113.7 | 416766.0 | 1197615.4 | 176827.2 | 145510.9 | 17326.3 | 69333.5 |
|  | **SJ10** | 53550.0 | 40801.9 | 331361.9 | 38255.3 | 3090116.2 | 127004.1 | 219455.9 | 3455.5 | 73241.9 |
|  | **SJ24** | 192063.8 | 30078.8 | 186572.3 | 288487.3 | 1149792.9 | 328024.2 | 13603.2 | 1298.9 | 153974.0 |
|  | **SJ30** | 33923.2 | 9383.3 | 22777.0 | 110916.2 | 2486849.2 | 216587.4 | 45117.0 | 9155.0 | 238967.6 |
| **Japan** | **SJ80** | 90734.9 | 58611.9 | 112726.4 | 160954.6 | 6109756.9 | 265663.7 | 1355691.6 | 28029.9 | 152972.6 |
| **China** | **SJ67** | 26469.4 | 25619.4 | 51172.5 | 884731.0 | 903444.3 | 7224.7 | 965152.6 | 15332.1 | 184987.3 |
|  | **SJ69** | 6631.6 | 6801.8 | 8988.8 | 54894.8 | 573441.4 | 6982.5 | 2311283.9 | 9630.5 | 76338.0 |
| **Taiwan** | **SJ37** | 25018.4 | 19736.8 | 44546.1 | 365794.1 | 10561635.7 | 214207.2 | 584339.3 | 1901.7 | 63874.9 |
|  | **SJ39** | 53800.5 | 3392.4 | 9672.7 | 342182.7 | 903131.1 | 199698.0 | 243038.6 | 7213.7 | 41889.5 |
| **Thailand** | **SJ86** | 43326.0 | 9596.6 | 18334.1 | 931858.7 | 1680592.9 | 177610.3 | 1478645.0 | 28811.4 | 64856.5 |
|  | **SJ87** | 2513.0 | 14786.9 | 32696.1 | 771624.1 | 2416548.2 | 139959.3 | 164524.0 | 32081.4 | 124107.8 |
|  | **SJ97** | 14512.7 | 5317.4 | 39136.4 | 498719.1 | 404285.9 | 146461.0 | 79516.6 | 25605.0 | 41572.5 |
|  | **SJ109** | 50658.8 | 12549.8 | 17408.9 | 1462247.9 | 1130378.5 | 69697.5 | 147669.0 | 48084.7 | 153259.8 |
| **India** | **SJ159** | 2968.8 | 4231.2 | 94825.4 | 361692.6 | 2212469.9 | 149337.8 | 50207.8 | 3575.5 | 333989.7 |
| **USA** | **SJ43** | 7541.9 | 5375.6 | 26358.0 | 69817.3 | 704786.0 | 50940.1 | 92172.1 | 6056.1 | 323341.7 |
|  | **SJ50** | 38463.8 | 5433.4 | 84066.1 | 356611.1 | 546437.6 | 334798.3 | 1107733.3 | 16559.6 | 63489.9 |
|  | **SJ262** | 28626.3 | 16805.0 | 15643.9 | 346881.1 | 14703353.8 | 185851.6 | 609314.5 | 16583.7 | 107864.4 |
| **Europe** | **SJ62** | 11321.6 | 15852.0 | 30481.9 | 805082.3 | 1300563.5 | 68537.1 | 1148054.1 | 24300.2 | 92259.3 |
|  | **SJ65** | 41168.9 | 27626.4 | 52696.3 | 933540.6 | 24545264.4 | 15232.7 | 644661.3 | 46452.1 | 201544.0 |
| **Etc.** | **SJ46** | 17766.4 | 21424.5 | 104561.9 | 69817.3 | 651481.0 | 224367.2 | 92696.9 | 24978.4 | 57299.8 |

Relative abundance values of significantly discriminant metabolites (VIP > 0.7) in twenty cucumber lines based on PLS-DA model (Figure 2E). HS-SPME-GC-TOF-MS, headspace-solid phase microextraction gas chromatography time-of-flight mass spectrometry; VIP, variable importance projection; PLS-DA, partial least squares-discriminant analysis. **p*-value < 0.05, ^d^N.I., Non-identified

**Table S6. Differential primary metabolites identified by GC-TOF-MS in the peel of three cucumbers (SJ24, SJ62, and SJ109)**

| **No.** | **Tentative Identification^a^** | **GC-TOF-MS** | | | | |
| --- | --- | --- | --- | --- | --- | --- |
|  |  | **Ret (min)^b^** | **Unique mass(m/z)** | **MS fragment pattern(m/z)** | **TMS** | **ID** |
| *Amino acids & amines* | | | | | | |
| 1 | Alanine | 5.77 | 116 | 73,116,147,59,75,117,77,74,103,100,58 | 2 | Lib/STD |
| 2 | Valine* | 6.99 | 218 | 73,144,147,59,218,75,100,74,145,72,103 | 2 | Lib/STD |
| 3 | Isoleucine | 7.76 | 158 | 73,158,74,218,100,59,159,147,58,86,103 | 2 | Lib/STD |
| 4 | Glycine | 7.89 | 174 | 73,174,86,147,59,100,175,133,248,74,75 | 3 | Lib/STD |
| 5 | Serine | 8.38 | 204 | 73,204,218,100,75,147,59,116,74,205,77 | 3 | Lib/STD |
| 6 | Threonine | 8.63 | 219 | 73,57,117,101,219,218,147,75,100,74,129 | 3 | Lib/STD |
| 7 | Aspartic acid | 9.39 | 232 | 73,232,100,75,147,74,218,117,59,233,133 | 3 | Lib/STD |
| 8 | 5-Oxoproline | 9.83 | 156 | 156,73,157,258,230,158,74,122,259,231,58 | 2 | Lib/STD |
| 9 | GABA* | 9.85 | 304 | 73,174,147,75,86,59,100,175,74,304,133 | 3 | Lib/STD |
| 10 | Phenylalanine* | 10.66 | 218 | 73,218,192,100,75,147,74,91,219,59,120 | 2 | Lib/STD |
| 11 | Tryptophan | 14.66 | 202 | 73,202,203,74,291,204,59,130,147,145,103 | 3 | Lib/STD |
| *Organic acids* | | | | | | |
| 12 | Pyruvic acid | 6.20 | 133 | 73,147,133,100,59,72,220,148,74,235,89,75 | 2 | Lib/STD |
| 13 | Succinic acid* | 7.91 | 247 | 147,73,75,55,56,148,77,247,149,129,74 | 2 | Lib/STD |
| 14 | Propanoic acid* | 8.11 | 189 | 73,147,189,73,103,292,133,102,74,205,117,75 | 3 | Lib/MS |
| 15 | Malic acid | 9.50 | 133 | 73,147,55,75,74,133,59,101,148,117,149 | 3 | Lib/STD |
| *Sugars & Sugar alcohols* | | | | | | |
| 16 | Threonic acid* | 10.12 | 292 | 73,147,117,75,103,292,74,220,55,102,205 | 4 | Lib/STD |
| 17 | Glucose* | 12.66 | 160 | 73,103,147,160,74,117,133,89,59,75,129 | 5 | Lib/STD |
| 18 | Myo-Inositol* | 13.92 | 305 | 73,147,217,191,74,133,129,103,75,147,204 | 6 | Lib/STD |
| 19 | Carbohydrate (1) | 11.41 | 217 | 73,217,103,147,205,129,319,307,218,189,204 |  | Lib/MS |
| 20 | Carbohydrate (2) | 12.80 | 319 | 73,147,73,160,319,205,103,205,320,74,319 |  | Lib/MS |
| 21 | Carbohydrate (3)* | 12.96 | 307 | 73,147,217,103,205,307,74,129,117,133,218 |  | Lib/MS |
| 22 | Carbohydrate (5)* | 14.43 | 204 | 73,204,147,205,217,75,129,263,103,117,191 |  | Lib/MS |
| *Fatty acids* | | | | | | |
| 23 | Linoleic acid methyl ester* | 14.46 | 337 | 73, 117, 129, 74, 145, 147, 146, 132, 84 | 2 | Lib/MS |
| 24 | α-Linolenic acid* | 14.51 | 108 | 75, 79, 73, 67, 95, 93, 80, 108, 81, 91, 94 | 2 | Lib/MS |
| 25 | Stearic acid | 14.62 | 341 | 73, 117, 75, 132, 129, 55, 145, 341 | 2 | Lib/MS |
| *Etc.* | | | | | | |
| 26 | Hydroxylamine | 5.93 | 133 | 73,133,146,119,59,86,147,72,130,100,74,249 | 3 | Lib/STD |
| 27 | Uracil* | 8.19 | 241 | 73,245,147,241,99,75,256,255,113,246,143,100 | 2 | Lib/MS |
| *N.I.* | | | | | | |
| 28 | N.I. 1* | 6.34 | 86 | 86,75,73,87,74,188,146,103,70,86,61,57 |  |  |
| 29 | N.I. 2* | 6.57 | 86 | 86,70,75,73,69,87,74,146,57,188,103,56 |  |  |
| 30 | N.I. 3 | 9.95 | 155 | 155,73,156,147,257,74,75,157,59,229 |  |  |
| 31 | N.I. 4 | 10.47 | 274 | 69,70,142,376,276,58,348,245,230,171 |  |  |
| 32 | N.I. 5* | 11.26 | 260 | 73,260,55,128,217,147,129,261,218,100 |  |  |
| ^a^Identified metabolites based on the VIP value (> 0.7) from the PLS-DA model; ^b^Retention time; TMS, Trimethylsilyl; MS, mass spectrum compared with the National Institute of Standards and Technology (NIST) database and in-house libraries; STD, mass spectrum consistent with that of the standard compounds; **p*-value<0.05. | | | | | | |

**Table S7. Differential volatile organic compounds identified by HS-SPME-GC-TOF-MS in the peel of three cucumbers (SJ24, SJ62, and SJ109)**

| **No.** | **Tentative**  **Identification^a^** | **HS-SPME-GC-TOF-MS** | | | | | |  |
| --- | --- | --- | --- | --- | --- | --- | --- | --- |
|  |  | **Ret (min)^b^** | **Unique mass**  **(m/z)** | **MS fragment pattern(m/z)** | **CAS** | **Molecular Formula** | **Weight** | **ID** |
| *Aldehydes* | | | | | | | | |
| 1 | Propanal | 1.37 | 58 | 58,57,55,59,53,56,52,54,60 | 123-38-6 | C3H6O | 58 | Lib/MS^c^ |
| 2 | Pentanal* | 2.11 | 58 | 58,57,55,53,56,50,51,71,67 | 110-62-3 | C5H10O | 86 | Lib/MS |
| 3 | Hexanal* | 3.06 | 57 | 57,56,72,58,82,67,55,71,53 | 66-25-1 | C6H12O | 100 | Lib/MS |
| 4 | 2-Hexenal | 4.89 | 98 | 55,69,83,57,56,70,98,53,80 | 505-57-7 | C6H10O | 98 | Lib/MS |
| 5 | 2-Octenal* | 7.99 | 70 | 55,70,57,83,69,82,67,58,56 | 2548-87-0 | C8H14O | 126 | Lib/MS |
| 6 | 6-Nonenal* | 8.01 | 93 | 55,54,81,67,70,93,79,69,83,56 | 2277-19-2 | C9H16O | 140 | Lib/MS |
| 7 | 2,6-Nonadienal | 9.12 | 109 | 70, 69, 67, 79, 81, 68, 53, 95 | 557-48-2 | C9H14O | 138 | Lib/MS |
| 8 | Benzaldehyde | 9.25 | 105 | 77,105,106,95,51,81,50,79,53 | 100-52-7 | C7H6O | 106 | Lib/MS |
| 9 | 2,4-Nonadienal* | 11.59 | 138 | 81,67,53,82,54,55,83,79,68,138 | 5910-87-2 | C9H14O | 138 | Lib/MS |
| 10 | Tetradecanal | 14.18 | 82 | 57,55,82,67,68,69,81,96,56,71 | 124-25-4 | C14H28O | 212 | Lib/MS |
| 11 | Hexadecanal | 15.35 | 82 | 57,55,82,68,67,69,96,83,71,81 | 629-80-1 | C16H32O | 240 | Lib/MS |
| *Alcohols* | | | | | | | | |
| 12 | 3-Methyl-1-  butanol | 4.80 | 55 | 55,70,57,56,69,53,71,54,58 | 123-51-3 | C5H12O | 88 | Lib/MS |
| 13 | 1-Pentanol* | 6.11 | 97 | 55,70,97,93,111,62,77,166,392 | 71-41-0 | C5H12O | 88 | Lib/MS |
| 14 | 1-Hexanol | 6.85 | 56 | 56,55,69,57,84,54,53,73,70,51 | 111-27-3 | C6H14O | 102 | Lib/MS |
| 15 | 1-Octen-3-ol* | 8.28 | 57 | 57,72,55,58,85,68,81,67,53,99 | 3391-86-4 | C8H16O | 128 | Lib/MS |
| 16 | 3-Nonen-1-ol* | 11.33 | 68 | 55,68,67,81,69,54,82,57,95,56 | 10340-23-5 | C9H18O | 142 | Lib/MS |
| 17 | 6-Nonen-1-ol | 11.73 | 67 | 67,55,68,82,95,81,54,69,57,53 | 35854-86-5 | C9H18O | 142 | Lib/MS |
| *Etc.* | | | | | | | | |
| 18 | 2-Ethylfuran | 1.92 | 81 | 81,96,53,67,95,82,65,51,50,52 | 3208-16-0 | C6H8O | 96 | Lib/MS |
| 19 | β-Ionone* | 14.33 | 177 | 177,91,93,77,135,79,107,105 | 79-77-6 | C13H20O | 192 | Lib/MS |
| 20 | Octanoic acid | 15.57 | 101 | 60,73,55,101,87,84,61,85,69 | 124-07-2 | C8H16O2 | 144 | Lib/MS |
| *N.I.* | | | | | | | | |
| 21 | N.I. 1 | 2.64 | 70 | 70,69,71,55,50,57,56,68,53,51 | C4H6O | 70 | - | - |
| 22 | N.I. 2* | 11.50 | 68 | 68,57,69,55,97,84,53,56,51,79 | - | - | - | - |
| ^a^Identified volatile compounds based on variable importance projection(VIP) analysis with cut-off value of 0.7; ^b^ Retention time; ^c^MS, tentatively identified by mass spectra(comparison with Willy, Replib, Mainlib Library & HMDB, Pubchem database; ^d^N.I., Non-identified; **p*-value<0.05 | | | | | | | | |

**Table S8. Differential primary metabolites identified by GC-TOF-MS in the flesh of three cucumbers (SJ24, SJ62, and SJ109)**

| **No.** | **Tentative Identification^a^** | **GC-TOF-MS** | | | | |
| --- | --- | --- | --- | --- | --- | --- |
|  |  | **Ret (min)^b^** | **Unique mass(m/z)** | **MS fragment pattern(m/z)** | **TMS** | **ID** |
| *Amino acids & amines* | | | | | | |
| 1 | Alanine* | 5.77 | 116 | 73,116,147,59,75,117,77,74,103,100,58 | 2 | Lib/STD |
| 2 | Valine* | 6.99 | 218 | 73,144,147,59,218,75,100,74,145,72,103 | 2 | Lib/STD |
| 3 | Leucine* | 7.55 | 158 | 73,158,75,59,147,103,102,74,117,159,133 | 2 | Lib/STD |
| 4 | Isoleucine* | 7.76 | 158 | 73,158,74,218,100,59,159,147,58,86,103 | 2 | Lib/STD |
| 5 | Proline* | 7.81 | 142 | 142,73,143,147,74,75,59,216,144,72,70,66 | 2 | Lib/STD |
| 6 | Serine* | 8.38 | 204 | 73,204,218,100,75,147,59,116,74,205,77 | 3 | Lib/STD |
| 7 | Threonine* | 8.63 | 219 | 73,57,117,101,219,218,147,75,100,74,129 | 3 | Lib/STD |
| 8 | Aspartic acid* | 9.39 | 232 | 73,232,100,75,147,74,218,117,59,233,133 | 3 | Lib/STD |
| 9 | 5-Oxoproline* | 9.83 | 156 | 156,73,157,258,230,158,74,122,259,231,58 | 2 | Lib/STD |
| 10 | GABA* | 9.85 | 304 | 73,174,147,75,86,59,100,175,74,304,133 | 3 | Lib/STD |
| 11 | Asparagine | 10.96 | 116 | 73,116,132,231,75,147,141,74,188,100,117 | 3 | Lib/STD |
| 12 | Tyrosine | 12.87 | 218 | 73,218,100,219,147,74,75,179,220,280,133 | 3 | Lib/STD |
| 13 | Tryptophan* | 14.66 | 202 | 73,202,203,74,291,204,59,130,147,145,103 | 3 | Lib/STD |
| *Organic acids* | | | | | | |
| 14 | Pyruvic acid* | 6.20 | 133 | 73,147,133,100,59,72,220,148,74,235,89,75 | 2 | Lib/STD |
| 15 | Propanoic acid* | 8.11 | 189 | 73,147,189,73,103,292,133,102,74,205,117,75 | 3 | Lib/MS |
| 16 | Malic acid* | 9.50 | 133 | 73,147,55,75,74,133,59,101,148,117,149 | 3 | Lib/STD |
| *Sugars & Sugar alcohols* | | | | | | |
| 17 | Erythritol | 9.68 | 217 | 73,147,117,103,217,75,205,116,69,101,204 | 4 | Lib/MS |
| 18 | Threonic acid* | 10.12 | 292 | 73,147,117,75,103,292,74,220,55,102,205 | 4 | Lib/STD |
| 19 | Fructose* | 12.56 | 308 | 73,103,217,147,74,149,133,117,307,59,89 | 5 | Lib/STD |
| 20 | Glucose | 12.66 | 160 | 73,103,147,160,74,117,133,89,59,75,129 | 5 | Lib/STD |
| 21 | Myo-Inositol* | 13.92 | 305 | 73,147,217,191,74,133,129,103,75,147,204 | 6 | Lib/STD |
| 22 | Carbohydrate (1)* | 11.41 | 217 | 73,217,103,147,205,129,319,307,218,189,204 |  | Lib/MS |
| 23 | Carbohydrate (2)* | 12.80 | 319 | 73,147,73,160,319,205,103,205,320,74,319 |  | Lib/MS |
| 24 | Carbohydrate (4)* | 13.49 | 204 | 73,204,205,147,217,220,319,74,103,148,129 |  | Lib/MS |
| 25 | Carbohydrate (5)* | 14.43 | 204 | 73,204,147,205,217,75,129,263,103,117,191 |  | Lib/MS |
| *Fatty acids* | | | | | | |
| 26 | Palmitic acid | 13.50 | 313 | 73, 117, 75, 132, 313, 129, 55, 145, 131 | 2 | Lib/STD |
| 27 | Linoleic acid methyl ester* | 14.46 | 337 | 73, 117, 129, 74, 145, 147, 146, 132, 84 | 2 | Lib/MS |
| 28 | α-Linolenic acid* | 14.51 | 108 | 75, 79, 73, 67, 95, 93, 80, 108, 81, 91, 94 | 2 | Lib/MS |
| *Etc.* | | | | | | |
| 29 | Ethanolamine | 5.93 | 133 | 73,133,146,119,59,86,147,72,130,100,74,249 | 3 | Lib/STD |
| 30 | Uracil* | 8.19 | 241 | 73,245,147,241,99,75,256,255,113,246,143 | 2 | Lib/MS |
| *N.I.* | | | | | | |
| 31 | N.I. 1* | 6.34 | 86 | 86,75,73,87,74,188,146,103,70,86,61,57 |  |  |
| 32 | N.I. 2 | 6.57 | 86 | 86,70,75,73,69,87,74,146,57,188,103,56 |  |  |
| 33 | N.I. 3* | 9.95 | 155 | 155,73,156,147,257,74,75,157,59,229 |  |  |
| 34 | *N.I. 4** | 10.47 | 274 | 69,70,142,376,276,58,348,245,230,171 |  |  |
| 35 | N.I. 5* | 11.26 | 260 | 73,260,55,128,217,147,129,261,218,100 |  |  |
| ^a^Identified metabolites based on the VIP value (> 0.7) from the PLS-DA model; ^b^Retention time; TMS, Trimethylsilyl; MS, mass spectrum compared with the National Institute of Standards and Technology (NIST) database and in-house libraries; STD, mass spectrum consistent with that of the standard compounds; **p*-value<0.05. | | | | | | |

**Table S9. Differential volatile organic compounds identified by HS-SPME-GC-TOF-MS in the flesh of three cucumbers (SJ24, SJ62, and SJ109)**

| **No.** | **Tentative**  **Identification^a^** | **HS-SPME-GC-TOF-MS** | | | | | | |  |
| --- | --- | --- | --- | --- | --- | --- | --- | --- | --- |
|  |  | **Ret (min)^b^** | **Unique mass**  **(m/z)** | **MS fragment pattern(m/z)** | **CAS** | **Molecular Formula** | **Weight** | **ID** | |
| *Aldehydes* | | | | | | | | |  |
| 1 | Propanal* | 1.37 | 58 | 58,57,55,59,53,56,52,54,60 | 123-38-6 | C3H6O | 58 | Lib/MS^c^ | |
| 2 | Pentanal | 2.11 | 58 | 58,57,55,53,56,50,51,71,67,59 | 110-62-3 | C5H10O | 86 | Lib/MS | |
| 3 | Hexanal | 3.06 | 57 | 57,56,72,58,82,67,55,71,53,54 | 66-25-1 | C6H12O | 100 | Lib/MS | |
| 4 | 2-Hexenal | 4.89 | 98 | 55,69,83,57,56,70,98,53,80,54 | 505-57-7 | C6H10O | 98 | Lib/MS | |
| 5 | 2-Heptanal | 6.46 | 83 | 55,83,57,56,70,69,68,84,53,97 | 57266-86-1 | C7H12O | 112 | Lib/MS | |
| 6 | 2,6-Nonadienal | 9.12 | 109 | 70, 69, 67, 79, 81, 68, 53, 95 | 557-48-2 | C9H14O | 138 | Lib/MS | |
| 7 | 2,4-Nonadienal | 11.59 | 138 | 81,67,53,82,54,55,83,79,68,138 | 5910-87-2 | C9H14O | 138 | Lib/MS | |
| 8 | Tetradecanal | 14.18 | 82 | 57,55,82,67,68,69,81,96,56,71,83 | 124-25-4 | C14H28O | 212 | Lib/MS | |
| 9 | Hexadecanal | 15.35 | 82 | 57,55,82,68,67,69,96,83,71,81,56 | 629-80-1 | C16H32O | 240 | Lib/MS | |
| *Alcohols* | | | | | | | | |  |
| 10 | 3-Methyl-1-  butanol* | 4.80 | 55 | 55,70,57,56,69,53,71,54,58,50,51 | 123-51-3 | C5H12O | 88 | Lib/MS | |
| 11 | 1-Pentanol | 6.11 | 97 | 55,70,97,93,111,62,77,166,392 | 71-41-0 | C5H12O | 88 | Lib/MS | |
| 12 | 1-Hexanol | 6.85 | 56 | 56,55,69,57,84,54,53,73,70,51,67 | 111-27-3 | C6H14O | 102 | Lib/MS | |
| 13 | 3,6-Nonadien-1-ol | 12.12 | 93 | 67,79,93,55,91,77,81,68,53,54,65 | 56805-23-3 | C9H16O | 140 | Lib/MS | |
| *Etc.* | | | | | | | | |  |
| 14 | 2-Ethylfuran | 1.92 | 81 | 81,96,53,67,95,82,65,51,50,52,97 | 3208-16-0 | C6H8O | 96 | Lib/MS | |
| 15 | Toluene | 2.60 | 91 | 91,92,65,63,51,50,93,89,62,90,64 | 108-88-3 | C7H8 | 92 | Lib/MS | |
| *N.I.* | | | | | | | | |  |
| 16 | N.I. 1* | 2.64 | 70 | 70,69,71,55,50,57,56,68,53,51,52 | C4H6O | 70 | - | - | |
| 17 | N.I. 2 | 11.50 | 68 | 68,57,69,55,97,84,53,56,51,79,81 | - | - | - | - | |
| ^a^Identified volatile compounds based on variable importance projection(VIP) analysis with cut-off value of 0.7; ^b^ Retention time; ^c^MS, tentatively identified by mass spectra(comparison with Willy, Replib, Mainlib Library & HMDB, Pubchem database; ^d^N.I., Non-identified; **p*-value<0.05 | | | | | | | | | |
